# DNA tension-modulated translocation and loop extrusion by SMC complexes revealed by molecular dynamics simulations

**DOI:** 10.1093/nar/gkac268

**Published:** 2022-04-26

**Authors:** Stefanos K Nomidis, Enrico Carlon, Stephan Gruber, John F Marko

**Affiliations:** Laboratory for Soft Matter and Biophysics, KU Leuven, Celestijnenlaan 200D, 3001 Leuven, Belgium; Flemish Institute for Technological Research (VITO), Boeretang 200, B-2400 Mol, Belgium; Laboratory for Soft Matter and Biophysics, KU Leuven, Celestijnenlaan 200D, 3001 Leuven, Belgium; Départment de Microbiologie Fondamentale, Université de Lausanne, 1015 Lausanne, Switzerland; Department of Physics and Astronomy, and Department of Molecular Biosciences, Northwestern University, Evanston, Illinois 60208, USA

## Abstract

Structural Maintenance of Chromosomes (SMC) complexes play essential roles in genome organization across all domains of life. To determine how the activities of these large (≈50 nm) complexes are controlled by ATP binding and hydrolysis, we developed a molecular dynamics model that accounts for conformational motions of the SMC and DNA. The model combines DNA loop capture with an ATP-induced ‘power stroke’ to translocate the SMC complex along DNA. This process is sensitive to DNA tension: at low tension (0.1 pN), the model makes loop-capture steps of average 60 nm and up to 200 nm along DNA (larger than the complex itself), while at higher tension, a distinct inchworm-like translocation mode appears. By tethering DNA to an experimentally-observed additional binding site (‘safety belt’), the model SMC complex can perform loop extrusion (LE). The dependence of LE on DNA tension is distinct for fixed DNA tension vs. fixed DNA end points: LE reversal occurs above 0.5 pN for fixed tension, while LE stalling without reversal occurs at about 2 pN for fixed end points. Our model matches recent experimental results for condensin and cohesin, and makes testable predictions for how specific structural variations affect SMC function.

## INTRODUCTION

**Figure 1.**
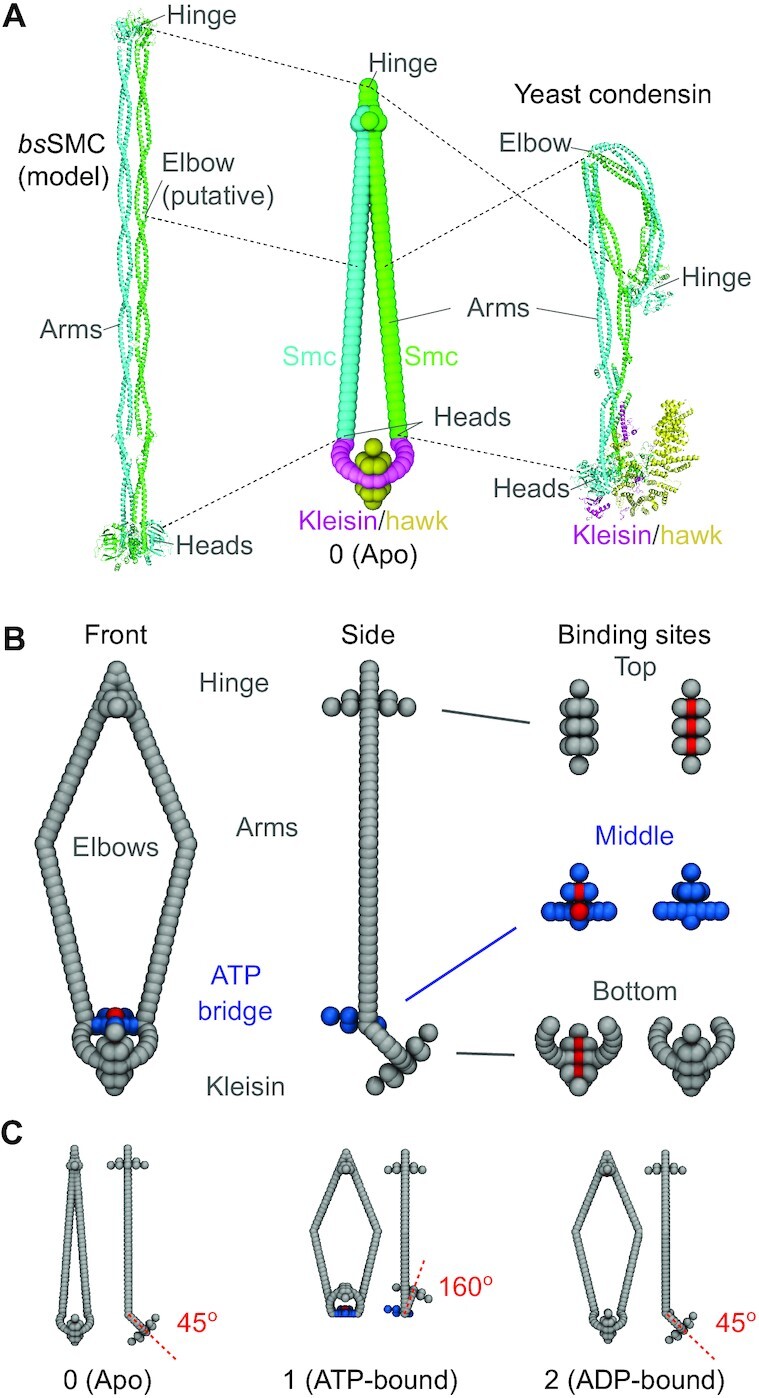
(**A**) SMCC structure in the ATP-unbound ‘apo’ or closed state. Left: Composite structural model of bsSMC (19), showing the two SMC proteins in cyan and green, the dimerization ‘hinge’ domain (top, PDB structure 4RSJ), the long coiled-coiled arms (PDB 5XG2 and 5NNV) with putative ‘elbow’ domain, and ATP-binding/ATPase ‘heads’ (PDB 5XEI). Additional subunits are shown in purple and. The two SMC proteins are adjacent, leaving little space between them. Right: Structural model of budding yeast condensin based on cryo-EM data (PDB 6YVU) (20) with the kleisin in purple and the additional ‘hawk’ subunit shown in yellow; for yeast condensin the coiled-coils are bent in the apo configuration, with the hinge domain near the lower part of the arms. Center: Simulation model of SMCC in the closed state, with SMC protein arms near one another. (**B**) Geometry of the simulation model, with coiled-coil arms ‘open’. A front and side view are shown on the left and in the middle, respectively. Beads that are repulsive and attractive to DNA are shown in light gray and red, respectively, while the DNA-repulsive ATP-bridge beads are indicated in blue. The ‘hinge’ domain, the ATPase-bridge domain, and the kleisin domain are shown separately on the right, with top and bottom views of the DNA-binding domains (bottom of ‘hinge’ domain, top of ‘ATPase bridge’ domain, top of domain, shown in red). Note that, this open SMCC configuration (open arms, ATPases bridged *and* kleisin at a 45-degree angle) is not an actual state of the model, but is used for illustration purposes. (**C**) The three structural states of the SMCC model, for no ATP bound or ‘apo’ (left), ATP bound ‘open’ state (center) with folded kleisin, and ADP-bound ‘ATPase bridge open’ state (right). In the apo state, the top, middle and bridge DNA binding sites are off; in the ATP-bound state the top, middle and bridge sites turn on; and in the ADP-bound state the middle, bridge and lower sites turn off.

Chromosomes in all living cells contain tremendously long DNA molecules, ranging in size from megabases (millimeters) in bacteria and other microbes, to gigabases (meters) in some animals and plants. Many lines of evidence have long pointed to DNA or chromatin loop formation as a fundamental organizing principle of chromosome folding, and it has now become clear that the Structural Maintenance of Chromosomes (SMC) protein complexes are key drivers of DNA looping (1–3). SMC complexes (SMCCs) are found in eukaryotes, bacteria and archaea, and possess a distinctive ring-like architecture. The rings include two SMC proteins with long, flexible regions that connect dimerization ‘hinge’ domains at one end to an ATP-binding domain ‘head’ at the other end (Figure 1A).

SMCCs undergo a series of conformational changes driven by ATP hydrolysis while interacting with DNA, leading to physical organization and folding of chromosomes. Cohesin and condensin are the two most studied eukaryotic SMCCs: condensin (4,5) folds and compacts chromosomes via formation of tightly-packed tandem loops (6–8), while cohesin (9) holds sister chromatids together during mitosis (10,11) and has also been found to play an important role in gene expression via formation of DNA loops (12,13). The bacterium *B. subtilis* possesses the bsSMC complex (14,15), and *E. coli* has the complex MukBEF (16–18). These bacterial SMCCs are structurally similar to condensin and cohesin, and fold chromosomes into highly-compacted, disentangled structures.

SMCCs are large (≈ 50 nm) and conformationally flexible. They are assembled as dimers of two SMC proteins which each have a dimerization domain at one end and an ATP-binding and hydrolysing domain at the other. The binding of the two SMC proteins at their dimerization domains forms the ‘hinge’ of the SMCC, while at the ATPase domain the SMCC has a more complex structure capable of large conformational changes. Figure 1A shows structural models for bsSMC and yeast condensin (19,20) along with our MD model, all for the ‘closed’ configuration with SMC arms adjacent to one another (Supplementary Table S1 of Supplementary Data lists corresponding structural features of these two SMCCs). This configuration corresponds to the ‘apo’ state where no ATP is bound.

A characteristic of all SMCCs is the presence of a kleisin subunit, which links the two SMC proteins to form a tripartite ring (21). *In vivo* experiments have established that DNA can be threaded through this protein ring (22–24). When ATP is bound by the ATPase domains of each of the two SMC proteins, the two heads can bind together to form a bridge, transforming the single ring of the SMCC to a two-compartment ring, consisting of a large ‘SMC’ compartment and a smaller ‘kleisin’ compartment (Figure 1B). The kleisin compartment is known to be able to trap DNA (25). ATP hydrolysis unlinks the ATPases, returning the ring to a single-compartment conformation, and then finally back to a conformation where the coiled-coil arms are adjacent to one another. Thus, the ATP binding, hydrolysis and release cycle is coupled to large-scale conformational changes of the SMCC.

A structural feature that varies among different SMCC species is the degree of folding of the coiled-coils at the elbow sites, to bring the hinge domain towards the kleisin region (e.g., Figure 1A, yeast condensin). Yeast condensin (20), cohesin (26,27) and *E. coli* MukBEF (26,28) are known to have this folding, while SMC5/6 (29,30) and *B. subtilis* bsSMC (31) are thought not to have it. As will be discussed, our model is not strongly dependent on the precise geometry of the complex, other than having the coiled-coils adjacent and the upper compartment ‘closed’ in the apo state, and having the upper compartment open when ATP is bound.

It has been hypothesized (1,2,6,7,12,13) that SMCCs are able to translocate along the DNA double helix (or along chromatin) in the manner of a molecular motor. Such a motor can perform active loop extrusion, e.g., by simply binding to one spot on the DNA while translocating (thought to be the case for yeast condensin (32)). Indeed, a series of experiments have observed ATP-dependent DNA compaction by condensin (33–35), translocation by condensin (36), and loop extrusion by condensin (37) and cohesin (38–41). The mechanism by which SMCCs perform these functions is unclear, although it must be based on ATP binding and hydrolysis, coupled to protein conformational change, and in turn coupled to DNA conformational fluctuation, which combine to produce translocation and loop extrusion.

Here, we present a coarse-grained molecular dynamics (MD) model which provides a generic description of SMCC activities. It is based on prior analytical work on SMCC translocation and loop extrusion (42), but the new simulation approach of this paper is able to take into account aspects of SMCC-DNA interactions which are difficult or impossible to deal with in a purely analytical framework, notably the synergy between conformational flexibilities of the SMC complex and the DNA it is moving along. The MD model is constructed from known features of SMCC-DNA interactions and the SMCC ATPase cycle, and contains enough detail to make a wide range of tests and predictions of SMCC behavior (Figure 1A-B). A number of models of SMCC function were proposed prior to our segment-capture model (42), based on mechanisms including ‘inchworm’ motions (43), a rotary step of a SMC subunit (44), and a non-motor based loop-ratchet (45). However, these works did not consider how the ATP-cycle-coupled conformational change of SMCCs and DNA random fluctuation work together to lead to active translocation and loop extrusion as observed experimentally, which is the focus of this paper.

The basic DNA segment-capture mechanism (42) qualitatively explains existing experiments on SMCC translocation along DNA, and subsequent theoretical work incorporating similar mechanisms involving binding at one DNA site with capture of a second, distant DNA (46,47) has led to concordant results. An alternative ‘scrunching’ model has also been proposed, based on the idea that DNA might be handed over from the hinge to the heads (or vice versa) via folding of the SMCC at an ‘elbow’ joint (36,48–50). However, elbow folding has not been observed for Smc-ScpAB and SMC5/6 (31,51,52). The positions of the elbow in condensin, cohesin and MukBEF are different, with hinge meeting arms, joints and heads, respectively, in those complexes, and the structure of the elbow is not conserved. This suggests elbow bending being a feature arising from divergent evolution, rather than being a feature fundamental to translocation and loop extrusion (20,26,53). While other simulation models can reproduce aspects of the experimental data (46,54), they are more coarse-grained than the model of the present paper. Our model is unique in including the known topological interaction between the SMCC tripartite protein ring and DNA, which is key to maintaining the translocation in one direction along DNA observed experimentally (54). Our model also describes the ATP binding and hydrolysis cycle and associated structural transitions of the SMCC, again based on experimental structural data.

Here, we report simulations of a MD realization of our analytical model that reveal new phenomena. We find that the flexibility of the SMC coiled-coils allows an alternative ‘inchworm’ mode of translocation to come into play, when the underlying DNA is under too much tension to permit DNA segment bending to easily occur (roughly for DNA tension above 1 pN). Anchoring of the DNA to the SMCC during translocation gives rise to loop extrusion, and we find that existing experiments observing loop extrusion on DNAs anchored at two fixed points (thus at *fixed DNA extension*) are also readily described, with loop extrusion persisting up to tensions of about 2 pN. Remarkably, we also find that, if a similar experiment is carried out at *fixed DNA tension*, different mechanoenzyme behavior is observed, with true stalling followed by ‘loop de-extrusion’, as force is increased beyond about 0.5 pN. Thanks to the role played by their distinctive structure in SMCC function, there are a number of conceivable modifications to SMCCs which should change their mechanical properties. We explore a few of these using our MD framework, predicting results for experiments on varied or mutated SMCCs.

## MATERIALS AND METHODS

### Model geometry

Our coarse-grained MD model is based on structural features common across SMCCs (19,42). The model was designed to be a minimal model incorporating the key geometrical and topological elements necessary for translocation, namely broken spatial and time symmetries. We will show that the behavior of the model is robust to appreciable parameter variation, but the key elements (the broken symmetries, the enzyme conformational cycle, and the topological coupling of the enzyme to DNA) are essential. It consists of individual rigid bodies (Figure 1B), which interact through bond, angle and dihedral potentials.

The geometrical details of the model have been chosen based on structural data for SMCCs as follows. Each of the two 50-nm-long coiled-coil arms consists of two straight segments, joined at a flexible ‘elbow’ (26), which gives them the ability to open and close (19,31). The two SMCs are connected at the top by a dimerization ‘hinge’. The hinge region of many SMCCs is known to contain a DNA-binding locus (55–61), although some studies indicate that having a DNA binding site at the hinge may not be essential (25). The SMCs can also be connected at their ATPase domain ‘heads’, via two bound ATPs, and by a kleisin subunit (19). The latter establishes the overall ring structure of the SMCC, and formation of the ATPase-ATP-ATPase ‘bridge’ (shown in blue in Figure 1B) can divide the ring into two compartments (25,28,62,63).

We will refer to the DNA-binding sites of the hinge, ATPase bridge and kleisin as *top*, *middle* and *bottom* binding sites, respectively (Figure 1B, right). We make special note of the middle site at the bottom of the upper compartment, which requires ATP binding and engagement. This is a highly-conserved feature across SMCCs, and has been found to be essential for translocation in bsSMC (25,27,63–66). We use energetic binding to model SMCC-DNA interactions, but to some degree these sites, in particular the lower one, may sterically trap or embrace DNA (25,63).

### SMCC structural states

The model SMCC has three distinct structural states, corresponding to the ATP-unbound (apo), ATP-bound, and ATP-hydrolyzed/ADP-bound states of the ATPase (Figure 1C) (42). In the *apo state* (0), the two arms and the upper compartment are closed, the ATP/ATPase bridge is open, the hinge DNA binding site is turned off and the lower compartment is folded by 45 degrees. In the *ATP-bound state* (1), which occurs upon ATP binding, the ATP bridge closes, the two arms open, and the lower compartment folds by an angle of 160 degrees. These conformational changes are supported by ultrastructural experimental data. Angular conformational changes of the lower compartment region of similar symmetry to those in our model and associated with ATP binding have been observed in cryo-EM studies of condensin and cohesin (20,27,28,53,66). In the case of yeast condensin this angular change involves the kleisin Brn1 and the Ycs4 HEAT-repeat subunit, and not the Ycg1 HEAT-repeat subunit (20,67). Partial opening and closing of the coiled-coils of the long SMC proteins have also been observed (20,67). Intriguingly, while high-speed AFM experiments have observed a ‘folding’ transition of condensin where the hinge moves to contact the Walker ATPase heads (48) this has not been observed in the recent cryo-EM experiments.

The opening of the arms makes the top DNA-binding site at the dimerization hinge accessible, which we model by turning on the top binding site of the open upper compartment. The same transition also turns on the middle DNA-binding site, at the lower part of the compartment. Finally, in the *ADP-bound state* (2), the two arms remain open, the bottom and middle binding sites are turned off, the folding angle of the kleisin is reduced back to 45 degrees and the ATPase bridge opens, and no longer separates the two compartments. Note that, the folding of the lower compartment is asymmetric, which is necessary for the motion of the SMCC to be directional.

The transition from each SMCC state to the next is modeled through changes of the equilibrium angles, which lead to opening/closure of the arms and folding of the kleisin. The bridge addition/removal is, likewise, modeled by the addition/removal of excluded volume interaction terms in the bridge beads. The transitions $0 \xrightarrow {k_{01}} 1 \xrightarrow {k_{12}} 2 \xrightarrow {k_{20}} 0$ take place at rates *k*_01_, *k*_12_ and *k*_20_, respectively. In practice, the simulation is maintained in a state *i* for a time interval *t*_*i*_, with *t*_*i*_ a random variable selected from an exponential distribution exp ( − *k*_*i*, mod(*i* + 1, 3)_*t*_*i*_). Within each state, the system evolved through ordinary MD with an implicit solvent Langevin thermostat. The configurations of Figure 1(C) are the minimal-energy states of the SMCC. Due to interactions with DNA and with the thermal environment, the SMCC structure fluctuates during simulations (see Supplementary Data Movies S1, S2 and S3). Parameters used to define the geometries and DNA-binding site dynamics for the different SMCC states are in Supplementary Table S2 and Supplementary Figure S1 of Supplementary Data.

### Translocation mechanism

**Figure 2.**
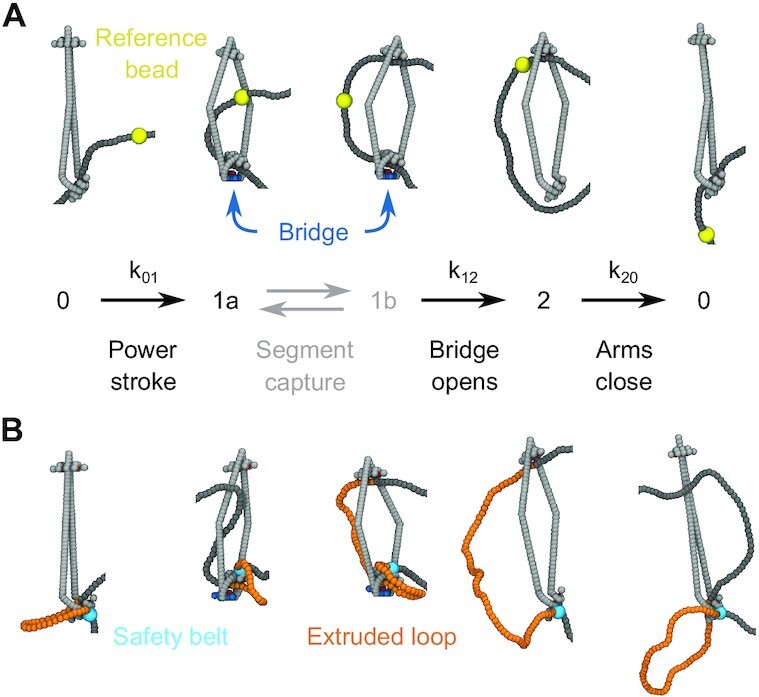
Sample MD simulation conformations of SMCC and DNA during (**A**) translocation and (**B**) loop extrusion. For translocation and loop extrusion the SMCC undergoes the same type of conformational changes, cycling through the three states labeled 0, 1 and 2 (Figure 1C). In the transition from 0 to 1 the kleisin folds and the ATPase bridge forms, directing the DNA inside the SMCC upper compartment. In the transition to 2 the bridged ATPases separate and the DNA detaches from the now-nonexistent bottom binding site on the SMCC. The closure of the arms pushes the DNA back to the bottom of the SMCC at the end of the cycle. The net effect is the translocation of DNA, shown here by the displacement of a reference bead (yellow). In the simulations, backward steps can be observed, particularly at higher DNA tensions (see text). In the loop extrusion case (**B**) the interaction DNA/SMCC is as in (**A**), but one site of DNA is permanently attached to the exterior of the kleisin subunit (cyan bead indicates this ‘safety belt’ binding site), hence the translocation along the DNA extrudes a loop (yellow).

In our model, the SMCC translocates along DNA via a segment-capture mechanism (42), as illustrated in Figure 2A. DNA is threaded through the lower compartment and bound at the bottom binding site. This is the apo state, with the two arms closed and the ATPase bridge open. The passage of DNA through the tripartite ring has been established in topology-sensing experiments on a range of SMCCs (22,23,25,28,68).

Upon transitioning to the ATP-bound state, the lower compartment folds, bringing DNA close to the middle binding site, to which it can efficiently bind (state 1a). This plays the role of a power stroke, as it forcibly pushes a DNA segment into the upper compartment. Notably, a recent cryo-EM structure of MukBEF has revealed that DNA is bound to that SMCC in the manner shown in state 1a, with the DNA passing through the asymetrically bent lower compartment (28). Subsequently, conformational fluctuations can lead distal DNA to bind to the upper binding site (state 1b), thus ‘capturing’ a bent DNA segment (one could describe this captured segment as a small loop, but we will avoid that to better distinguish it from the larger, extruded DNA loops).

Following segment capture upon ATP hydrolysis, the ATPase bridge opens, and conformational changes in the lower compartment lead to the middle and bottom binding sites being no longer active. Given that the DNA sequestration in the lower compartment may be due to steric trapping (25,63), the release of DNA from the lower site could be a natural consequence of the opening of the bridge. With the bridge gone, the captured DNA segment can release bending stress by escaping through the bottom of the complex. Finally, the SMCC returns to its apo state, with the closing of the two arms entropically pushing the DNA back to the now-reactivated bottom binding site. This sequence of states, ending with transport of the distal end of the captured segment from the top to the bottom of the SMCC protein loop, results in translocation of the SMCC along the DNA (indicated by the yellow reference DNA bead, Figure 2A).

An essential feature of this reaction cycle is that the DNA segment is never passed through the tripartite SMC-SMC-kleisin protein ring (this may occur during loading, but not during the translocation cycle). In addition to being supported by experimental observations of topological linkage of SMCCs to DNA mentioned above, this topological feature is key to the high degree of processivity and conserved translocation directionality of SMCCs along their left-right symmetric dsDNA substrate observed experimentally (36). Without this linkage, SMCCs would be unable to preserve their direction of translocation along dsDNA (42,67). We also emphasize that in this model the SMCC transitions are decoupled from DNA motion, in the sense that steps in the protein conformational cycle are not contingent on e.g., the presence of DNA bound at particular SMCC loci. At present there is no evidence for such ‘synchronizing’ interactions although they could certainly be added to the model. The absence of this type of synchronization in the present model means that it is possible for the segment capture process to fail, i.e., for futile SMCC cycles to occur.

### Loop-extrusion mechanism

Given translocation, loop extrusion may occur in a variety of ways (42,69). In this paper, we assume that a DNA segment (cyan bead in Figure 2B) is attached to the exterior of the kleisin subunit, as is thought to be accomplished by the ‘safety belt’ of yeast condensin. This DNA binding site is formed by Ycg1 and part of Brn1 (32), distinct from the regions of the Brn1 and Ycs4 involved in the ATP-driven angular conformational change observed in cryo-EM experiments (20,67). Combining safety-belt site binding with the threading of DNA through the lower compartment leads to formation of a DNA loop. In this situation, translocation, as described above, leads to asymmetric loop extrusion, as the unattached end of the loop translocates along DNA. We note that, establishment of this mode of loop extrusion requires passage of the DNA through the SMC-kleisin protein ring (*topological loading*), possibly involving opening of a SMC-kleisin ‘gate’ (66,70). We explore alternate loop-extrusion mechanisms compatible with our translocation model in the Discussion.

### Computational methods

#### MD

All simulations were performed in LAMMPS (71), using a standard velocity-Verlet integration scheme coupled to a Langevin thermostat. The simulation temperature was 300 K, with a damping parameter of 0.5 ns, while the simulation timestep was 0.2 pLsec (we use the units of Lsec to refer to LAMMPS time to distinguish from real experimental time measured in seconds).

#### DNA

DNA was modeled as a semiflexible bead-and-spring polymer (dark gray beads in Figure 2). Each bead represented 5 bp with a mass of 0.005 ag (3 kDa), while successive beads were connected through finitely-extensible springs, with a rest length of 1.7 nm. We emphasize that our simulations results are numerically independent of the bead masses due to the fact that the SMCC and DNA dynamics of interest here are on a much longer time scale than the relaxation time of bead velocity. A demonstration of the independence of kinetics of the DNA on bead mass is included in Supplementary Figure S5. The DNA stiffness was modeled through angle interactions among three successive beads, yielding a persistence length of 50 nm. Excluded-volume interactions were taken into account by means of truncated and shifted Lennard-Jones interactions, with an interaction distance of 3.5 nm, i.e., the effective DNA diameter at physiological salt conditions (72). A total of 301 beads were used, corresponding to a 1.5-kbp sequence with a contour length of 510 nm.

#### SMCC

The SMCC consisted of 7 rigid bodies (Figure 1B): four coiled-coil segments, the top binding site, the ATP bridge together with the middle binding site, and the kleisin subunit together with the bottom binding site. These interacted with each other by means of bond, angle and dihedral potentials. Each bead had a diameter 1.5 nm, and the total mass of the SMCC was chosen to be 0.25 ag (150 kDa). The coiled-coil arms were made of two connected, 25-nm-long, straight segments, interacting through a harmonic angle potential of 30 *k*_B_*T*/rad^2^ stiffness. The upper binding site consisted of a total of 17 beads. Three of these were chosen to be attractive to DNA (red beads), while the rest of them were repulsive (light gray beads). This ensured that DNA could only bind from a single direction. The ATP bridge was made of 2 attractive (red) and 6 repulsive beads (blue) attached to a 7.5-nm-long, straight segment (blue). The kleisin subunit consisted of 3 attractive (red) and 14 repulsive beads (light gray) attached to a circular arc of radius 7 nm (light gray). The top binding site, the ATP bridge and the kleisin subunit were kept aligned by means of bond and dihedral interactions.

The attraction of DNA by the binding sites was modeled with a truncated and shifted Lennard-Jones interaction, with a potential depth of 3.2 *k*_B_*T* for each top- and middle-binding-site bead, and 11 *k*_B_*T* for the bottom-site beads. In order to control the angle between the ATP bridge and the lower half of each arm, harmonic angle potentials were used, with a stiffness of 100 *k*_B_*T*/rad^2^. The two arms were made bendable by introducing similar harmonic angle potentials, with a stiffness of 30 *k*_B_*T*/rad^2^. The asymmetric folding of the kleisin subunit was achieved through two harmonic dihedral interactions, with stiffness constants of 60 *k*_B_*T*/rad^2^ and 100 *k*_B_*T*/rad^2^. The SMCC cycled stochastically through the three different states, apo (0), ATP-bound (1) and ADP-bound (2), with mean dwell times of τ_01_ = 0.4 μLsec, τ_12_ = 1.6 μLsec and τ_20_ = 0.4 μLsec (LAMMPS time units). Instantaneous dwell times were drawn from exponential distributions.

For the determination of captured DNA loop sizes we identified where the upper SMCC compartment encircled DNA. In particular, we located the DNA bead closest to the center of mass of the upper compartment, and also the one closest to the top binding site, and compared the two distances. This determined whether the system was in state 1a or 1b, i.e., DNA was closer to the center of mass or the top binding site, respectively. The loop end was associated with the smallest-proximity DNA bead.

## RESULTS

### Translocation

**Figure 3.**
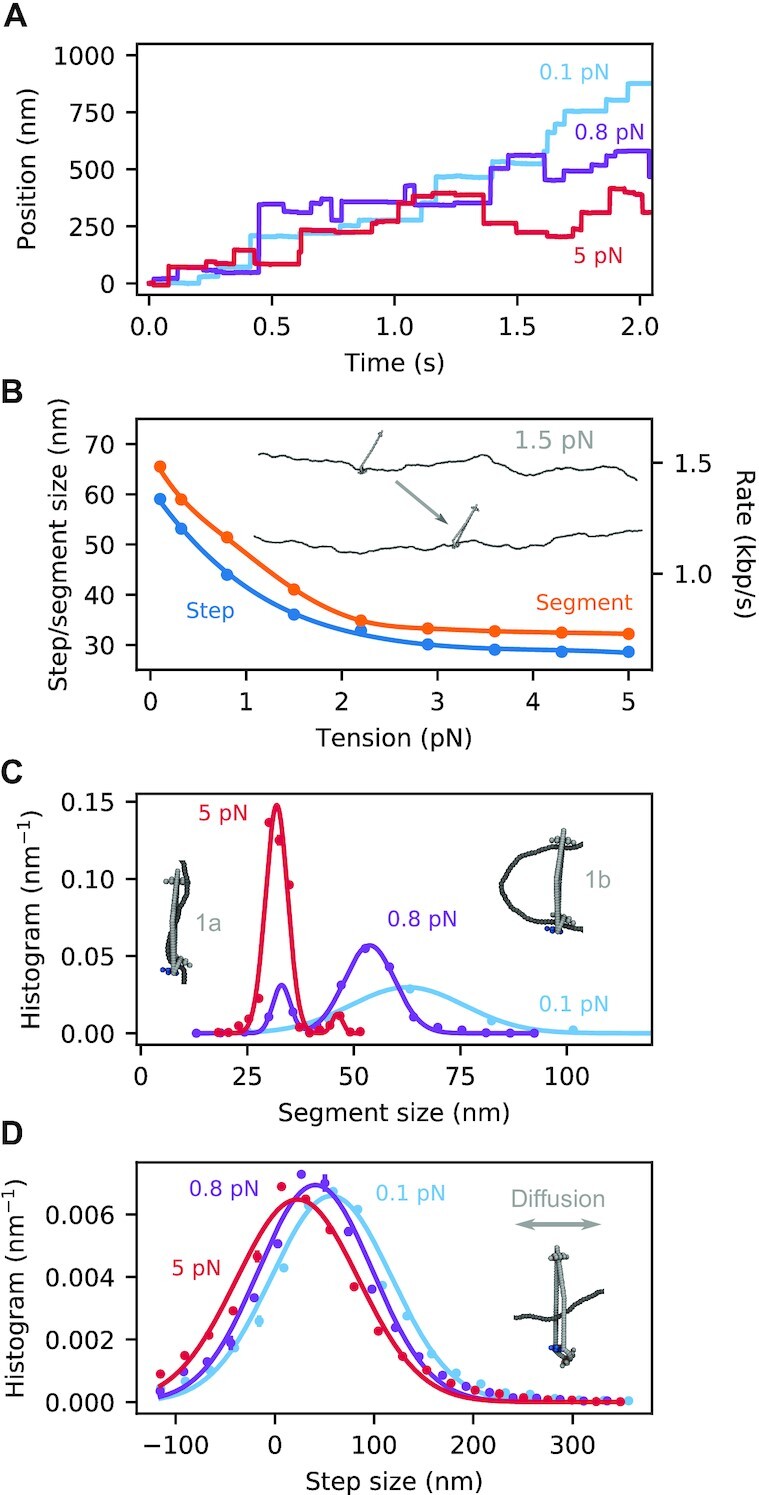
DNA translocation by SMCC. (**A**) Representative traces of the SMC position along the DNA track for three selected values of the DNA tension. These were obtained by stacking together independent SMC cycles under the same initial conditions. (**B**) Mean step size (blue points) and captured segment length (ATP-bound state 1 in Figure 2A, shown with orange points) dependence on DNA tension. Solid lines are spline interpolations, while the right axis converts the mean step size to translocation rate, using the conversion factor derived in Figure 4. The inset shows the before-and-after configurations for a typical cycle at 1.5 pN. (**C**) Segment-size distribution for some selected tensions (normalization is chosen so that the distribution integrates to 1). The solid lines are single- and double-Gaussian fits to the data; for the double-peak distribution the left and right peaks correspond to substates 1a and 1b respectively (inset snapshots; see text). (**D**) Step-size distribution for some selected tensions. Notice the difference in the length scale and width of the distributions between (**C**) and (**D**). This is a result of the SMCC diffusing during the transition 2 → 0 (see attached snapshot). Standard errors are plotted for all averaged quantities and in most cases are smaller than the data points.

We performed MD simulations for translocation of an SMCC along DNA under varying DNA tension, i.e., with stretching force applied to the two DNA ends (see Supplementary Data, Movies S1 and S2). Figure 3A shows time traces of SMCC positions along the DNA track for three different DNA tensions. The position is here defined as the coarse-grained DNA bead with minimal distance from the SMCC bottom site. This quantity changes slowly when the SMCC is in the states 0 and 1, while it shifts abruptly with the closing of the arms during the transition 2 → 0. At the smallest tension in Figure 3A (0.1 pN) the steps shown are all forward, while an increasing number of backward steps are observed as the tension increases. The timescale shown in the figure is based on experimental data of Ref. (37) and is applied by rescaling our simulation timescale to have each cycle correspond to 0.13 sec (see below). Figure 3B shows the observed translocation step size (blue points), averaged over many repeated cycles. As expected, the translocation slows down with increasing values of DNA tension, since it requires substantial bending of DNA (see Figure 2A), which becomes progressively unfavorable as the latter gets stretched. Interestingly, the translocation of the SMCC does not halt at large DNA tensions, but instead reaches a plateau value of about 30 nm per cycle. This is due to the asymmetric folding of the lower compartment in the ATP-bound state, which may be viewed as a power stroke (state 1a in Figure 2A), and is essentially unaffected by the physiologically-relevant forces (<10 pN) considered here.

We also measured the mean captured segment length in the ATP-bound state of the SMC complex (orange points in Figure 3B). The data show a similar trend to the translocation steps, but are shifted to larger values by an approximately-constant distance. This indicates that DNA slippage occurs in the ADP-bound state, when the SMCC is not bound to DNA at all (transition 2 → 0 in Figure 2A), by an amount essentially insensitive to DNA tension. The size of the captured segment is comparable to the persistence length of DNA (50 nm) and can be well described by a simple free-energy-minimization model (Supplementary Data, Supplementary Figure S2).

Figure 3C shows the distribution of captured segment lengths in the ATP-bound state for a few values of DNA tension (all distributions are normalized so that their integrals are 1, hence the units of step size distribution of nm^−1^). We note the appearance of two distinct peaks at intermediate and high forces (i.e., above about 0.2 pN). These peaks are indicative of two distinct substates, the relative population of which is tension dependent. The main distinction between the two substates is the attachment of a DNA segment to the top binding site (see snapshots), which is directly controlled by entropic fluctuations. The attached state is suppressed by high DNA tension, which favors state 1a over 1b. Movies S1 and S2 in Supplementary Data show a typical translocation cycle at low and high DNA tension, respectively, and highlight this distinction.

For comparison, the respective distributions of the translocation step size are plotted in Figure 3D, and are substantially wider than their ATP-bound captured segment counterparts, with a tension-independent spread. This implies the existence of appreciable random diffusion of the SMCC during the cycle, and in particular during the transition from the ADP-bound state back to the apo state (snapshot in Figure 3D), when the SMCC is not directly attached to DNA. As a result, the SMCC can perform some backward steps (negative tails in Figure 3D), but it executes a net directed motion after averaging over many whole cycles. The translocation direction is solely determined by the orientation of the SMCC upon its topological loading, since the DNA track is left-right symmetric. The left-right asymmetry, necessary for processive motor activity, arises from the folding of the kleisin protein. Processivity is maintained by the topological linkage between the SMCC and the DNA (Figure 2A).

### Loop extrusion

Next, we investigated the loop extrusion process by the SMCC model, based on the safety belt mechanism (Figure 2B). We distinguish between two cases, depending on the origin of tension in DNA. In the first case, the tension is externally fixed, by applying a stretching force to the ends of the molecule. This can be realized with single-molecule micromanipulation techniques, such as optical or magnetic tweezers. In the second case, it is the end points of DNA that are externally fixed, which has been experimentally demonstrated e.g., using DNAs attached at both ends to a surface (36–38). As loop extrusion progresses, the nonextruded DNA is gradually stretched, leading to a corresponding increase in its tension.

### Fixed tension

**Figure 4.**
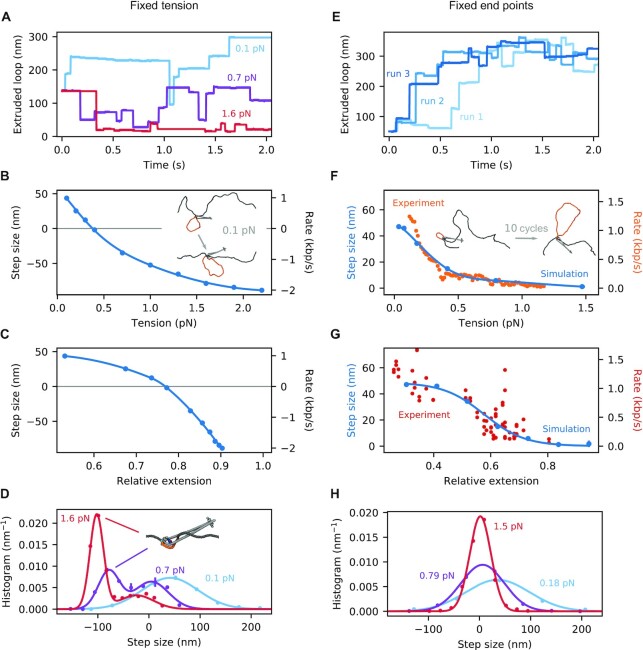
Loop extrusion of DNA by an SMCC under fixed DNA tension (A-D) and end-point distance (E-H). (**A**) Representative time traces of extruded DNA length by SMC for three selected values of tension. (**B**) Loop extrusion step size as a function of DNA applied tension. The solid line is a spline interpolation of the simulation data (points). The inset shows the before-and-after configurations for a typical cycle at 0.1 pN. (**C**) Same data plotted against the corresponding mean relative extension. The solid line is again a spline interpolation of the simulation data (points). (**D**) Distribution of loop extrusion steps for some selected tensions. The solid lines are single- and double-Gaussian fits to the data (points). The attached snapshot is a representative configuration associated with the second peak at high tensions. **(E)** Representative time traces of extruded DNA length by SMC for three independent simulation runs. **(F)** Loop extrusion step size as a function of the mean tension. The latter is computed from the mean net force exerted on the DNA ends. The solid line is a spline interpolation of the simulation data (blue points). For comparison, the experimental data of Ref. (37) are also shown with (orange points). The latter were originally obtained as a loop extrusion rate (right axis), so we transformed them into mean step size (left axis) by multiplying them with a factor of 44.2 s·nm/kbp, implying a cycle duration of 0.13 s. The inset shows some typical before-and-after configurations after the lapse of 10 cycles. **(G)** Same data plotted against the corresponding mean relative extension. The solid line is a fit of a logistic function. For comparison, the experimental data of Ref. (37) are also shown as red points. **(H)** Distribution of loop extrusion steps for some selected tensions. The solid lines are single-Gaussian fits to the data (points). Standard errors are plotted for all averaged quantities and in most cases are smaller than the data points.

Figure 4A shows representative time series of extruded loop size during simulations at constant tension, starting from an initial condition of a loop of 400 bp (about 136 nm) captured by the SMCC. The time series are extremely stochastic, and indicate that an initially-loaded loop grows for small tensions, and shrinks for larger ones. Averaging over many such runs leads to a smooth dependence of the mean loop extrusion step size, as a function of applied tension (Figure 4B), with extruded steps initially becoming smaller with increasing DNA tension, as for translocation (Figure 3B).

However, unlike translocation, for extrusion, sufficiently large tensions lead to the motor performing backward steps (above 0.4 pN), leading to a net shrinkage of the extruded loop. This takes place during the ADP-bound phase of the cycle (state 2 in Figure 2B), during which the loop can exchange length with the rest of the DNA. Notably, the amount of slippage per cycle is dependent on the loop size at the beginning of the cycle. A comparison among different initial loop sizes is shown in Supplementary Figure S3B of Supplementary Data; for tensions below stalling and slippage, the average step size converges with increasing initial loop size.

Figure 4C shows the same data, as a function of the mean relative extension. The latter is computed using the known equilibrium relation between force and extension (73). The step size distribution for some selected values of the tension is shown in Figure 4D, and is bimodal at high tensions. Analysis of the emergent second peaks reveals the presence of ‘imperfect cycles’: The nontethered part of the DNA does not return to the bottom binding site at the end of the cycle, but instead remains in the upper compartment. This further exposes the loop to the external tension, leading to substantial loop shrinkage, i.e., strongly negative steps.

### Fixed end-to-end extension

Next, we performed molecular dynamics simulation of loop extrusion at fixed end-to-end DNA extension, as studied experimentally (37). We started these simulations at a relative extension (ratio of end-to-end distance divided by DNA contour length outside the SMCC) of 25%, corresponding to a DNA tension of roughly 0.04 pN. The SMCC performed a total of 10 loop extrusion cycles per simulation run (see Movie S3 of Supplementary Data). Figure 4E shows a few representative extruded loop sizes versus time and one sees much less noisy behavior than in the fixed tension case (compare Figure 4A), with saturation of the extruded loop size at roughly 300 nm (900 bp) size as DNA tension rises to the point where it limits capture of loops by the enzyme.

Figure 4F shows the mean loop extrusion step size as a function of the average tension (blue points). This is obtained by a direct computation of the DNA tension after each cycle, and subsequent binning of the data. Note that, contrary to the fixed-tension case (Figure 4B), fixing the end points puts a hard limit on the DNA length that can be extruded, and loop extrusion halts at high tension with a large extruded loop, without reversal. The value of the mean step size at low tension (47 nm per cycle) is in quantitative agreement with that for the fixed-tension case (Figure 4B).

Ganji *et al.* have experimentally measured the rate at which condensin extrudes loops in DNA with fixed end points, using a single-molecule assay (37). These experiments were used to set the timescales for all of our simulations, using the fact that the ATP hydrolysis step (1b → 2 in Figure 2) is rate limiting in our simulations, with the other steps being in pre-equilibrium. This allowed us to rescale our cycle time so as to put our simulation and the experimental data on the same time scale. We found that choosing a cycle duration of 0.13 s resulted in an excellent correspondence between our simulations and experimental data (orange points in Figure 4F). Figure 4G shows data from the same simulations and experiments, plotted versus relative DNA extension (end-to-end distance over nonextruded DNA length). Again we find good agreement between simulation and experiment, together with the gradual slowing down of the motor at large DNA extension (high tension). This single time rescaling is used to set all the timescales for our MD results in Figures 3 and 4.

Figure 4H shows the distribution of the mean loop extrusion step size for some selected mean tensions. In all cases, the data can be well fitted with a single Gaussian peak. Comparison between Figures 4D and 4H further highlight the equivalence of the two situations (i.e., fixed tension and end-to-end extension) for low DNA tension/extension. Recent observations of step size distributions for yeast condensin (54) are in good agreement with our results.

### Varying the model parameters

**Figure 5.**
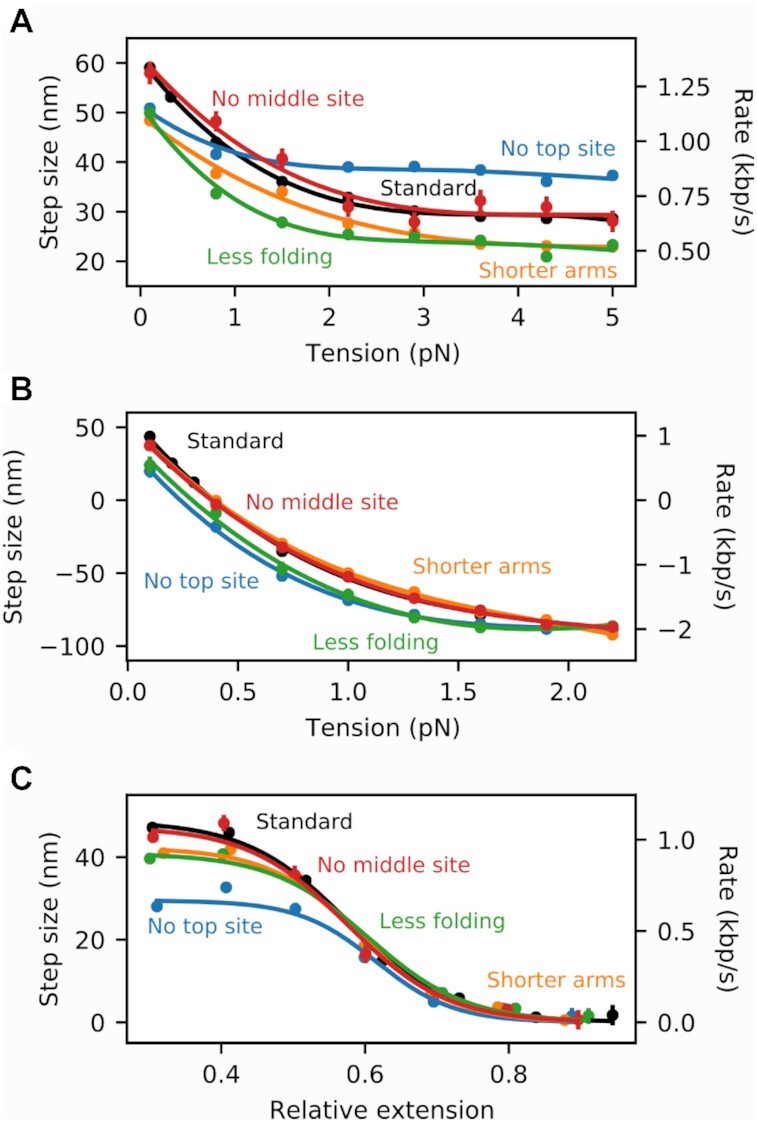
Comparison among different sets of model parameters for (**A**) translocation, (**B**) loop extrusion under fixed tension and (**C**) loop extrusion under fixed end-point distance. The black points correspond to the parameters used throughout this work (standard), the blue points to a deactivation of the top binding site (no top site), the red points to a deactivation of the middle binding site (no middle site), the orange points to a shortening of the coiled-coil arms from 50 nm to 40 nm (shorter arms) and the green points to a reduction of the folding angle of the lower compartment in the ATP-bound state from 160 degrees to 130 degrees (less folding). Standard errors are plotted for all averaged quantities and in most cases are smaller than the data points.

In order to investigate how the behavior of the motor depends on the model details, we performed additional translocation, fixed-tension and fixed-end-point loop extrusion simulations for different model parameters (Figure 5, panels A, B and C, respectively). More specifically, we deactivated the top binding site throughout the whole cycle, and found that the motor could still operate, suggesting that the existence of the top binding site is not a necessary element in the model (blue points). Interestingly, the translocation of the motor was found to be more efficient at high tension. This is likely due to the 2 → 0 transition being faster in that case, since DNA needs to travel a shorter distance until it reaches the bottom binding site. Similarly, the SMC complex was found to be insensitive to a deactivation of the middle binding site (red points).

Additionally, we reduced the length of the coiled-coil arms from 50 nm down to 40 nm, and observed little to no difference (Figure 5, orange points). We also reduced the folding angle of the lower compartment in the ATP-bound state from 160 to 130 degrees, which slightly slowed down the motor but did not keep it from translocating (green points). Finally, making the elbows in the SMC arms completely flexible (zero bending stiffness) did not keep translocation from occurring, although it proceeded via somewhat smaller steps at low force (Supplementary Figure S3A, Supplementary Data). This emphasizes that conformational and mechanical details of the upper compartment are less important than the breaking of symmetry and topological separation of upper and lower compartments that occurs upon ATP binding. We conclude that the MD model motor function is qualitatively insensitive to a variety of modifications, and that it is likely applicable to a range of SMCCs. The quantitative predictions of the specific modifications we have examined amount to predictions for realizable SMCC mutation experiments.

## DISCUSSION

The results presented above comprise a detailed analysis of the segment-capture model for SMC translocation and loop extrusion (Figure 2) (19,42). The numerical nature of our model circumvents analytical limitations (42), and allows us to make a number of predictions for future experiments.

### Translocation

Our model predicts the DNA-tension dependence of translocation by SMCCs (Figure 3B), with the translocation rate dropping as DNA tension is increased through about 1 pN. Prior analytical modeling (42) did not fully explore the effect of a possible power stroke, in the form of kleisin folding. In our MD simulations, this allows translocation to proceed even when the DNA is tightly stretched, via an ‘inchworm’-like mechanism (smaller peak in captured segment size distribution, Figure 3C). The drop to a plateau translocation rate with increasing force (Figure 3B) is the signature of translocation occurring through two distinct mechanisms (two peaks in Figure 3C), namely DNA bending and segment capture at lower forces vs. ‘inchworming’ (possibly involving SMC bending) at high forces. We note that substrate tension-feedback control of SMC function has been seen in other models (74), an effect similar to our observation of a change in translocation mechanism as DNA tension is varied.

To date, no translocation experiments at controlled tension have been carried out. Observation of translocation by yeast condensin observed a velocity of 60 bp/s for DNA tension estimated to be 0.3 pN (36). This is substantially slower than the respective estimate of about 1.2 kbp/s from our MD model (Figure 3B), the cycling rate of which was set using loop-extrusion experiments (37). This deviation may, thus, reflect a large variability in the motor efficiency, depending on the precise experimental conditions.

In addition to the tension-velocity behavior, the MD model makes clear predictions for the size distribution of the DNA segment capture (Figure 3C) (which, for the inchworm mode, corresponds to essentially the length of the SMCC), as well as for the translocation step (Figure 3D). The model shows that the relatively narrow captured DNA segment size is broadened by diffusion into a more smeared-out DNA step size distribution. The smearing is a result of the entropic transport of DNA from the top binding site back to the bottom one following ATP hydrolysis (Figure 2 2→0), during which diffusion of the SMCC along the DNA can freely occur. Cohesin has been observed to diffuse on DNA (75) in experiments lacking the motor-processivity protein NIPBL (38).

The model’s key transition, which breaks left-right symmetry and provides a power stroke, is head engagement and folding of the lower compartment (Figure 2 0→1a), guiding the DNA to the middle binding site on top of the Walker ATPase heads (Figure 2 1a). This leads to formation of a DNA segment in the upper compartment (Figure 2 1a↔1b). A remarkable result is that the top binding site is dispensable, in that translocation and loop extrusion persist without it (Figure 5A-C, blue curves). In accord with this, a recent genetic experiment on bsSMC mutated away putative DNA-binding residues in the upper ‘hinge’ domain, with little effect on translocation and loop extrusion *in vivo* (25). We have also observed that the middle binding site on the ATPase bridge is not required for translocation and loop extrusion (Figure 5A-C, red curves).

Our observation of a lack of necessity for the upper-compartment DNA-binding site may help to explain the variability in the hinge-proximal DNA-binding across SMCCs. While the presence of a positively-charged ‘channel’ (with a, so far, poorly-understood function) along with a positively-charged surface on the hinge are conserved (58), the precise location of basic residues on the hinge surface varies appreciably across SMCCs (56,57,60,61). This variability may reflect our result that the top binding site is not crucial, and is involved in fine-tuning of SMCC function. Our model enzyme cycle does require DNA release from the top site following arm refolding, and we can expect the top site to be relatively weak, so as to have its interaction with DNA disrupted by the return to the apo state.

Current experiments and Figure 3 do not consider the effect of a load force, examining DNA translocation only as a function of DNA tension. It would be informative to additionally apply a direct load to the enzyme, and to examine translocation velocity as a function of both DNA tension and enzyme load force. This could be realized with a combination of three applied forces using, e.g., triple force-controlled optical tweezers, with forces *f*_tension_ and *f*_tension_ − *f*_load_ at the two ends of the DNA, and *f*_load_ applied to the SMCC (42). A recent study observed translocation for cohesin at a velocity of 0.4 kbp/s against a buffer flow (38). The latter may introduce both DNA tension (by stretching) and a load force on the SMCC, although in an imperfectly quantitatively-controlled fashion.

### Loop extrusion: fixed end points vs. fixed tension

The MD model quantitatively describes compaction rates observed in experiments on loop extrusion at fixed DNA end-point distance (37) (Figure 4F,G), and additionally predicts step sizes and their distributions (Figure 4H). The tension in the DNA builds up as a loop is extruded, and there comes a point at which the enzyme stalls. Due to this externally-induced tension-extrusion coupling, the loop can never shrink, and thus the MD rate vs. tension is always positive, asymptotically approaching zero for large forces, as observed experimentally (Figure 4F,G).

The positivity of compaction rate with fixed end points is in stark contrast to the situation for fixed DNA tension, where there is a well-defined stall tension, beyond which an initially-extruded loop will start shrinking (Figure 4B,C). Indeed, our MD model is eventually forced to run in reverse, taking negative steps of well-defined size (Figure 4D). Evidence for reversal of SMCC loop extrusion/translocation by force exists, in the form of Hi-C data from *B. subtilis* consistent with bsSMC-RNAP collisions forcing bsSMC backwards along DNA (76). Experiments on loop extrusion vs. controlled DNA tension would provide further insight into how SMCCs on DNA can be pushed around by other enzymes. *In vivo* one can imagine loop extrusion being opposed by both fixed endpoint and fixed tension restraints, the former being relevant to a chromosomal domain, which is defined by binding to solid cellular structures at two distant points, and the latter being relevant to molecular motors, such as polymerases, which might act to generate tension in a DNA segment.

### Head engagement power stroke

In the MD model, ATP binding and head engagement are associated with a conformational change of the SMCC, which facilitates segment capture from one side of the enzyme (Figure 2 0→1). Such a symmetry-breaking event is necessary for the translocation to be directional, otherwise DNA segments would be captured with equal efficiency from both directions, and the enzyme would move randomly left and right along its unpolarized DNA track. This conformational change might be directly observable in an experiment that monitors the enzyme itself, e.g., by monitoring the distance between ATPase heads directly, or the effect of head engagement on overall conformation of the enzyme.

In our SMCC model, head engagement and kleisin folding move the lower edge of the enzyme by a distance of roughly 10 nm (vertical distance moved by lower edge between states 0 and 1 in Figure 2 0→1). In our model, this transition can actually be observed in terms of a small contraction in the flanking DNA (Supplementary Figure S4, Supplementary Data), although to actually observe this it is likely that a quite short DNA will have to be used (our MD simulations use a total of 1.5 kb).

By applying sufficient force against this conformational change, one might be able to keep it from occurring, providing measurement of the enzyme power stroke. The threshold to overpower the conformational change would likely be a force in the vicinity of 10 pN, i.e., the force scale associated with breaking noncovalent biomolecule-biomolecule interactions, or the stalling of molecular motors. The origin of this force scale is in the range of forces required to change conformation of or to unfold proteins by force; in this case, one is acting against the force driven by the binding of ATP to link together the two Walker ATPase subunits. This force can be estimated by dividing the free energy associated with ATP hydrolysis (≈20 *k*_B_*T*) per ATP) by the conformational change (≈3 nm), which leads to ≈25 pN. Our results show that this force is associated with the stalling of translocation but is only indirectly related to that for loop extrusion (Figure 3B vs. Figure 4F). Recent observations of large ATP hydrolysis-independent contractions of SMCC-DNA complexes (54) are likely looking at the DNA segment capture process rather than protein conformational change.

### SMCC conformation and flexibility

A feature of the model that we have explored is the effect of SMC coiled-coil ‘arm’ flexibility on SMCC translocation and loop extrusion. We have found that making SMC arm joints completely flexible does not abrogate translocation (Supplementary Figure S3A, Supplementary Data), and in fact eliminates the need for DNA bending, thus likely facilitating translocation at high DNA tensions. This may be important, given that there are observations of rather extreme SMC arm flexibility for condensin (48,77), although recent cryo-EM images of precisely the same type of condensin (20) suggest conformational properties similar to what we have assumed here. However, essentially the same motor function should result from variations on the protein-ring closure mechanism presumed here, for example an ATP-dependent ‘collapse’ of the protein ring (54).

The main features needed for SMCCs to translocate in the manner described by our model are the folding/breaking of symmetry in the lower compartment, and the formation of separated upper and lower compartments, both a result of ATP binding. The key elements of our model are largely topological: passage of DNA through the protein ring, and the ATP-dependent division of the ring into upper and lower compartments. Models of SMC function which do not incorporate the topological nature of SMCC-DNA interaction will have weak directional processivity due to the left-right symmetry of duplex DNA. Given this, changes to SMCs which reduce the area of opening of the coiled-coil upper compartment will likely tend to impairment of translocation and loop extrusion.

We have found that DNA binding interactions, apart from some mechanism to hold on to DNA in the lower compartment, are largely dispensable, with elimination of the upper and middle sites not significantly changing translocation and loop extrusion (Figure 5). Our model may overestimate this robustness due to the strong symmetry-breaking folding of the kleisin, which allows MD simulation of loop capture on a computationally-manageable timescale, but which may also lead to an underestimation of the importance of the loop-capturing DNA binding sites. Modification of the strength of the DNA binding sites is also a reasonable way to model changes in univalent salt concentration, with weaker interactions corresponding to higher salt concentration. However, precise quantitative calibration of this in our coarse-grained description is problematic.

### Loading topology and symmetry of loop extrusion

**Figure 6.**
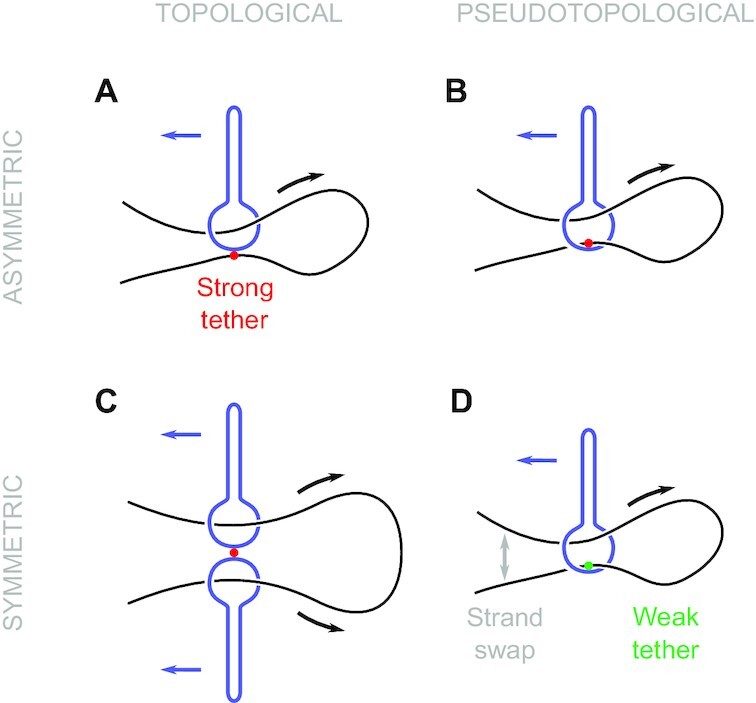
Different loop extrusion mechanisms, categorized according to loading mechanism (vertical) and symmetry of loop extrusion (horizontal). (**A**) Permanently attaching DNA to the exterior of the SMCC (safety belt, marked with red) leads to asymmetric loop extrusion. This requires DNA threading through the lower compartment, and is the mechanism used in this work. (**B**) Moving the tethering point to the interior of the SMCC does not require opening of the protein ring, but rather an insertion of a DNA hairpin. (**C**) Two permanently-joined SMCCs translocating in opposite directions can symmetrically extrude loops. (**D**) If the tether in the interior of the SMCC is weak, such that DNA detachment and strand swap can take place, the same pseudotopological model as in (**B**) can now symmetrically extrude loops.

As discussed earlier, translocation is the fundamental function of SMCCs that underlies all modes of loop extrusion. Depending on the SMCC, it appears that different modes do occur, with distinct loading mechanisms and symmetry of loop extrusion (Figure 6). For example, it has been suggested that yeast condensin translocation and loop extrusion require passage of the DNA through a transient opening of the SMCC protein ring (33). For this SMCC, the apparent anchoring of the DNA to the outside of the ring (safety belt) requires topological loading, and leads to asymmetric loop extrusion (Figure 6A), formally the mode used in our MD model.

In principle, our MD model may also be applied to the case where the DNA is bound *inside* the SMC-kleisin ring (Figure 6B). While this is geometrically identical to the external safety-belt scheme (the relocation of the tethered DNA strand from the outside to the inside of the kleisin is inconsequential to the cycle of Figure 2B), there is a key topological difference: no opening of the protein ring is required to initiate the loop extrusion process, since the DNA can be bent into a hairpin and then inserted into the SMC-kleisin ring (pseudotopological loading) (42). As long as DNA remains tethered in the interior of the SMC-kleisin ring, this mechanism generates one-sided, asymmetric loop extrusion, similar to the external safety belt. Recent DNA mapping studies suggest that condensin may bind DNA in this manner (67).

A third possibility is a simple variation of the above, where the tethering of DNA is weak enough to allow transient detachment and reattachment (Figure 6D). Since two DNA segments are in close proximity in the interior of the SMCC protein loop, a strand swap can take place, possibly via facilitated dissociation (likely a strong effect under the strong confinement of two DNA strands in the SMCC lower compartment) (78). If this occurs relatively slowly compared to the cycling time, the result will be progressive extrusion on both sides of the loop i.e., symmetric pseudotopological loop extrusion, as indeed has been observed for cohesin (38). Each strand swap event translates to a change in which side of the loop DNA segments are captured from, which might be detectable given sufficient temporal resolution.

Finally, a fourth possible ‘dimerized translocator’ can result from the coupling of two SMCCs together (Figure 6C), each translocating to an opposite direction, so as to accomplish symmetric loop extrusion at double the velocity of a single translocator (42). This scenario requires topological loading of DNA segments into both of the dimerized SMC-kleisin rings. Evidence exists for dimerization of the *E. coli* SMCC MukBEF *in vivo* (79–81), as well as for ATP-dependent compaction activity by oligomerized condensins *in vitro* (34,39). Examination of all of these scenarios for loop extrusion using MD simulations of the sort we have reported here would be desirable. However, they all present a higher degree of computational difficulty than that of the current paper, with the dimerized translocator being particularly extreme in this regard.

This suggests a rule: single SMCCs that require topological loading *must* asymmetrically extrude loops (at least between successive protein loop-opening events), while ones that load pseudotopologically *can* perform symmetric loop extrusion (cohesin). A recent study indicates that condensin from metaphase *Xenopus* egg extracts drives more asymmetric loop extrusion than does cohesin from interphase extracts (40), consistent with condensin being topologically loaded, and (interphase) cohesin being pseudotopologically loaded. The precise rules for how loading and loop extrusion occur *in vivo* are likely regulated by factors that mediate SMCC protein loop opening (63,66).

### Obstacle bypass and formation of z-loops

SMCCs have recently been observed to be able to ‘bypass’ an obstacle during loop extrusion, in the sense that loop extrusion has been observed to continue following the encounter of obstacle and SMCC (82). This appears inconsistent with the long-established topological linkage between DNA and tripartite SMCC protein rings (22,23), and with segment-capture models (19,42) which incorporate this feature to obtain processivity, translocation and loop extrusion (19,42). However, a resolution of this apparent conflict has been proposed for the segment-capture model (67) (note that Figure 6 of Ref. (67) is topologically equivalent to the variant of loop extrusion for the segment-capture model shown in Figure 7B in Ref. (42) and Figure 6B of this paper, all with an initial loop of DNA passing twice through the lower compartment, a subsequent ATP-binding-driven ‘power stroke’ guiding upstream DNA segment capture in the upper compartment, and then ATP hydrolysis and Walker ATPase separation which causes enlargement of the initial DNA loop by merging it with the captured DNA segment (83)).

In the new experiments (82), a relatively large colloidal particle was tethered to a specific point along the DNA, leading to a collision between an SMCC and the obstacle. Supplementary Figure S6A-B illustrates this for translocation: when all the DNA ‘downstream’ of the obstacle (Supplementary Figure S6A, purple) is pulled through the SMCC, a point is reached where the obstacle blocks further translocation (Supplementary Figure S6B). The key insight of Ref. (67) is that in this obstacle-blocked state, DNA on the *upstream* side of the obstacle (Supplementary Figure S6, green DNA) can still be captured by the SMCC leading to extrusion of a loop with the obstacle effectively acting as the ‘loop anchor’ (Supplementary Figure S6C-F). In the experiments of Ref. (82), the use of a flexible PEG linker between the DNA and the large bead-obstacle may facilitate this process, but similar ‘obstacle bypass’ can likely occur past some objects that are directly bound to the DNA, so long as the downstream DNA is able to be captured by the middle (bridge) binding site. A consequence of this that is biologically relevant is that obstacles that are able to block SMCC translocation may act as loop-extrusion initiation sites, causing compaction of downstream DNA into an extruded loop.

Supplementary Figure S6G-H shows this process including anchoring of the upstream (purple) DNA to form a loop (loop 1) which cannot grow beyond the obstacle attachment point. Capture of the downstream DNA (green) leads to formation of a second loop (loop 2). In this state, if one applies a force to the obstacle (e.g., via fluid flow in the case where the obstacle is a relatively large colloidal particle) the topology is such that a third loop can be ‘pulled out’ of the SMCC (Supplementary Figure S6H, loop 3).

The same general ideas can be applied to the case where the obstacle is a loop-extruding SMCC along the same DNA as a second loop-extruding SMCC (Supplementary Figure S7A). As the ‘outer’ SMCC (right, condensin b) approaches the ‘inner’ one (left, a), DNA downstream (red) of the inner SMC can be captured in the outer one (Supplementary Figure S7B), leading to extrusion of a new loop of downstream DNA (Supplementary Figure S7C). When rearranged without topology change, this is easily deformed into a ‘z-loop’ structure as observed in recent experiments with colliding SMCCs (Supplementary Figure S7D) (84). These structures can be undone using force (e.g., fluid flow), by pulling the newly extruded loop back out of the z-loop (Supplementary Figure S7E). This general scheme is also consistent with Hi-C maps indicating traversal of one SMCC by another *in vivo* which have been interpreted in terms of z-loop formation (85).

## DATA AVAILABILITY

The LAMMPS parameters that define the MD model and the scripts used to generate simulation data are available at https://github.com/sknomidis/SMC_LAMMPS. All numeric data are available on request to the corresponding author.

## Supplementary Material

gkac268_Supplemental_Files
