# Supplementary material for: DNA tension-modulated translocation and loop extrusion by SMC complexes revealed by molecular dynamics simulations
Source: Nucleic Acids Res. 2022 Apr 26;50(9):4974–87. doi: 10.1093/nar/gkac268 (PMC9122525; doi:10.1093/nar/gkac268)
Supplement: gkac268_Supplemental_Files [file gkac268_supplemental_files.zip › supplemental2.pdf]

Supplementary Data for

DNA tension-modulated translocation and loop extrusion by SMC complexes revealed by molecular dynamics simulations

Stefanos K. Nomidis, Enrico Carlon, Stephan Gruber, John F. Marko

Corresponding Author: John F. Marko
E-mail: john-marko@northwestern.edu

This PDF file includes:

Supplementary text
Figs. S1 to S7
Tables S1 to S2
Legends for Movies S1 to S3
SI References

Other supplementary data for this manuscript:

Movies S1 to S3

Supporting Information Text

DNA binding sites

Table S1. DNA binding sites assumed in model along with corresponding protein regions and supporting experimental data. Left column lists first the model nomenclature (top, middle, bottom, anchor/safety belt) and the related structural features. Middle column lists protein subunits, amino acid residues involved in DNA binding, and relevant references, for the *B. subtilis* SMC. Right column lists the protein subunits, residue numbers, and references for yeast condensin.

DNA binding site		Bsu Smc-ScpAB			Yeast condensin		
		Subunit	Residues	Reference	Subunit	Residues	Reference
top	hinge	Smc	K666, K667, K668	(1) (2)	Smc2<br>Smc4	positively charged<br>surface patch observed	(3) (4) (5) (6)
middle	ATP-heads	Smc	R54, K60, R120<br>K122, R153	(2)	Smc2<br>Smc4	site likely based on wide<br>conservation (Rad50 and *Bsu* Smc)	(2) (7)
bottom	kleisin-kite/hawk	ScpAB	Steric binding (putative)		Ycs4	site likely based on<br>similarity to cohesin Scc3	(8)
anchor	safety-belt		Unknown		Ycg1<br>Brn1	K70, K71, K849<br>K409, R411, K456	(9)<br>(9)

Coarse-grained SMCC model

Table S2. Summary of the SMCC simulation parameters used for modeling the three different states. The angles are defined as shown in Fig. S1 and are expressed in degrees, while stiffnesses in units of $k_{\mathrm{B}}T/\mathrm{rad}^2$. The curly brackets indicate the values used to produce some of the variations of Fig. 5 of the main text.

	APO	ATP/bound	ADP/bound
**Bridge**	OFF	ON	OFF
**Top site**	OFF	ON {OFF}	ON {OFF}
**Middle site**	OFF	ON {OFF}	OFF
**Lower site**	ON	ON	OFF
Rest angles			
θ_0	$81°$	$130°$	$130°$
ϕ_0	$45°$	$160°\{130°\}$	$160°\{130°\}$
ψ_0	$180°$	$180°$	$180°$
Stiffnesses			
K_θ	100	100	100
K_ϕ	60	60	60
K_ψ	30	30	30

Captured segment length

The translocation and loop-extrusion function of the SMCC is based upon a segment-capture process, which takes place during its ATP-bound state (Fig. 1C,D of main text). Here, we present an estimate of the segment length *vs.* the DNA tension, based on a free energy minimization. We assume that the SMCC stays sufficiently long at that state, so that we can treat the system as being at equilibrium. As the captured segment length (Fig. 2A of main text) is comparable to the persistence length of DNA (50 nm), one may neglect the former's conformational fluctuations, and focus on its minimal-energy shape (below we refer to the bent DNA segment captured during a translocation cycle as a DNA "loop"; we note that this is distinct from the larger *extruded* loop). The total free energy of the system is then given by

$$F = \varepsilon_{\mathrm{loop}}(L) + (L_{\mathrm{total}} - L)g(f), \quad [1]$$

L_{total} is the total DNA length, $\varepsilon_{\mathrm{loop}}(L)$ the minimal energy for a loop of length L (blue line in Fig. S2A) and $g(f)$ the free energy per unit length of a linear (*i.e.,* unlooped) DNA under a tension f (black line in Fig. S2A). To obtain the loop length for the given DNA tension, one needs to minimize Eq. (1) with respect to L. Note that, we have omitted length-independent terms in Eq. (1), as they do not affect our calculation.

Both $g(f)$ and $\varepsilon_{\mathrm{loop}}$ can be estimated based on the wormlike chain model, which treats DNA as a semiflexible polymer. The free energy density of a stretched DNA can be obtained by integrating its relative DNA extension with respect to the tension. In the high-force limit, this is simply (10)

$$g(f) = -f\left(1 - \sqrt{\frac{k_{\mathrm{B}}T}{Af}}\right), \quad [2]$$

where $A = 50$ nm is the persistence length of DNA and $k_{\rm B}T \approx 4.1$ pN·nm is the thermal energy at room temperature. Since, here, we are also interested in intermediate forces (down to 0.1 pN), we will numerically estimate $g(f)$ using an interpolation formula for DNA extension (11), see Fig. S2C.

The loop can be parametrized with an angle θ, denoting the orientation of the tangent with respect to the x-axis (Fig. S2A), while its energy is given by

$$\varepsilon_{\rm loop} = \frac{Ak_{\rm B}T}{2}\int_0^L \left(\frac{{\rm d}\theta}{{\rm d}s}\right)^2 {\rm d}s, \tag{3}$$

where s is the arclength coordinate. We wish to minimize this energy with respect to $\theta(s)$, under the constraints $\theta(0) = \pi/2$ and $\theta(L) = 3\pi/2$, *i.e.,* by keeping the tangents antiparallel at the loop ends points, which resembles the action of the SMCC on DNA in the ATP-bound state (state 1b in Fig. 1C,D of main text). A simple variational solution can be obtained through a circle-line approximation (12, 13), as illustrated in Fig. S2B. Loops of length $L < \pi d/2$ (with d the end-point distance) are approximated with two quadrants connected with a straight line, whereas longer loops by a semicircle extended with straight lines. The loop energy of such a circle-line model is

$$\varepsilon_{\rm loop}^{\rm CL} = \begin{cases} \dfrac{A\pi k_{\rm B}T}{L-d}\left(\dfrac{\pi}{2}-1\right), & \text{for} \quad L < \pi d/2 \\[2ex] \dfrac{A\pi k_{\rm B}T}{d}, & \text{else.} \end{cases} \tag{4}$$

The loop shape can also be obtained from a Fourier-series expansion of the curvature, ${\rm d}\theta/{\rm d}s$, as recently shown in Ref. 14 for closed and open loops with free boundary conditions. It turns out that, under fixed boundary conditions, $\theta(0)$ and $\theta(L)$, the Fourier series can be rewritten into the simple form (unpublished result)

$$\theta(s) = \pi - \frac{\pi^2}{\pi^2-8}\left[\left(c-\frac{4}{\pi}\right)\cos\left(\frac{\pi s}{L}\right) + \left(\frac{\pi}{2}-c\right)\left(1-\frac{2s}{L}\right)\right], \tag{5}$$

where the parameter c fixes the relative end-point distance, d/L, as follows

$$\int_0^1 \cos\left\{\frac{c-\pi/2}{1-8/\pi^2}\left[1-2\sigma-\cos(\pi\sigma)\right] - \frac{\pi}{2}\cos(\pi\sigma)\right\}{\rm d}\sigma = \frac{d}{L}. \tag{6}$$

Note that, $c = 4/\pi$ describes a semicircle, while $c = \pi/2$ a loop with a single Fourier component. Finally, substitution of Eq. (5) into Eq. (3) yields the energy

$$\varepsilon_{\rm loop} = \frac{A\pi^2 k_{\rm B}T}{4L}\left[c^2 + \frac{(c-\pi/2)^2}{\pi^2/8-1}\right]. \tag{7}$$

Figure S2D shows a comparison between Eq. (4) and Eq. (7) (dashed and solid lines, respectively), for varying loop size. Obviously, the exact calculation yields consistently-lower energies than the circle-line approximation, with the exception of the semicircle case, $L = \pi d/2$, where the two coincide. Moreover, smaller loops have a larger energy, due to their increased curvature. Note that, the circle-line energy is constant for $L > \pi d/2$, as any additional loop length is absorbed into the energetically-inconsequential straight segments.

One can now minimize Eq. (1) with respect L, using either Eq. (4) or Eq. (7), so as to obtain the minimal-energy loop size for fixed DNA tension, shown in Fig. S2E with dashed and solid green lines, respectively. For comparison, the loop-size MD data from Fig. 2A of main text are shown (blue points). At high DNA tension, the theory deviates from the simulation data, as partial segment capture starts taking place (state 1a in Fig. 3C of main text), which is not accounted for by this theory. Excluding these events from the calculation of the mean step size (orange points), reveals a good agreement with the theory. Note that, the loop end-point distance is the only parameter of the calculation, and it is fixed at $d = 35$ nm from the SMCC geometry.

DNA contraction during the SMC cycle

In the SMCC model, the upward folding of the kleisin moves the lower edge of the enzyme by a distance of approximately 10 nm. This is the vertical distance moved by lower edge when a transition between the states 0 and 1 takes place (see Fig. 2 of the main text). These transitions can actually be observed in simulations as small contractions in the flanking DNA, as illustrated in Fig. S4. When the SMCC is in the state 1, the end-to-end distance of the DNA is shorter than with SMCC in states 0 or 2. The difference is small and decreases at higher applied tension.

Testing the overdamped dynamics of DNA

In coarse-grained simulations with implicit solvent, the mass assigned to each coarse-grained bead should also take into account the correlated motion of the surrounding fluid. LAMMPS (15) integrates the equations of motion using a total force of the type $F = F_c + F_f + F_r$ with F_c a conservative force, $F_f = -mv/{\rm d_m}$ a viscous force and $F_r \propto \sqrt{\frac{k_B T m}{\Delta t\ {\rm d_m}}}$ a random force. In the previous expressions v is the velocity, ${\rm d_m}$ is the damping coefficient and Δt the integration timestep. The viscous and random

forces depend on the mass through the ratio $m/\mathrm{d_m}$, while the mass enters in the equations of motion $ma = F$ through the inertial term ma. Inertia has typically an effect on the short time scale dynamics, while it can be safely neglected at long times. In order to probe inertial effects we performed some test simulations on a our coarse-grained model of DNA by rescaling the mass and damping coefficients by the same factors so to keep the ratio $m/\mathrm{d_m}$ constant. We considered a loop of length $L = 136$ nm (400 bp) with fixed endpoint and probed the dynamics of the central monomer for which we calculated the mean squared displacement $\Delta r^2(t)$. Figure S5 shows plots of this quanitites for various values of bead mass of DNA, with m being the value reported in the main text. The excellent overlap of the data indicate that inertial effects are negligible beyond the timescale of 1 ns.

Obstacle bypass by SMCC

In a recent experimental study (16) a nanoparticle of about 200 nm in size was attached to a specific location on DNA via a PEG-linker. The nanoparticle is much larger in size than a SMCC protein ring (~ 50 nm). Loop extrusion was then observed to not necessarily stop when the SMCC pulls DNA in to the point where the loop base and SMCC meet the large obstacle. This observation was interpreted as proving that the SMCC can "bypass" the obstacle and therefore that the DNA cannot be inside the (crosslinked) tripartite protein ring. The observations are done using optical microscopy, with a maximum imaging resolution in the 100 nm range and one observes obstacle and DNA loop (bright dot indicating a random coil) in the same location, to optical resolution. The experiment cannot test whether the obstacle has passed through the protein ring. In the case that our model SMCC encounters such an impassable obstacle, our segment-capture mechanism can continue to operate as illustrated in Fig. S6. In conclusion, we believe that the recent experiments (16) do not invalidate the segment capture model discussed in this paper. It is certainly interesting to investigate quantitatively how the present SMCC model copes with obstacles of various sizes. This is beyond the scope of the present manuscript and will be left for future studies.

Condensins crossing one another

Recent experiments on interactions condensin-DNA showed, quite surprisingly, that two condensins can traverse one another creating so-called Z-loops, where three double-stranded DNA helices align in parallel with one condensin at each edge (17). Figure S7 shows a possible explanation of this phenomenon within the segment-capture model.

Summary of statistics used to obtain averages

All data was derived from simulations of strings of SMCC ATP cycles, which we refer to as "steps". Each step consisted of an average of 2.4 μLsec of simulation time (0.4 in the apo state, 1.6 in the ATP state, and 0.4 in the ADP state). Since the microscopic MD timestep was 0.2 pLsec, 1.2×10^7 MD steps were used to simulate each SMCC cycle (SMCC step).

Figure 3: 7997, 11226, 7838, 7599, 7659, 7826, 7500, 7891, 7821 SMCC steps were used for 0.1, 0.32, 0.8, 1.5, 2.2, 2.9, 3.6, 4.3, 5 pN, respectively.

Figure 4A-D: 996, 1337, 1345, 1004, 965, 1069, 1057, 964, 905, 903 steps were used for 0.1, 0.2, 0.3, 0.4, 0.7, 1, 1.3, 1.6, 1.9, 2.2 pN, respectively.

Figure 4E-H: A total of 35806 steps were used. These were binned as: 7395, 6767, 5609, 6471, 6785, 2640, 139 steps were used for relative extension 0.3, 0.41, 0.52, 0.62, 0.73, 0.84, 0.94, respectively.

Figure 5A red: 910, 989, 1024, 1032, 971, 944, 996, 957 steps were used for 0.1, 0.32, 0.8, 1.5, 2.2, 2.9, 3.6, 4.3, 5 pN, respectively.

Figure 5A blue: 4183, 4115, 4124, 4368, 4463, 4160, 4235, 4172 steps were used for 0.1, 0.32, 0.8, 1.5, 2.2, 2.9, 3.6, 4.3, 5 pN, respectively.

Figure 5A green: 3116, 3304, 3376, 3487, 3443, 3445, 3370, 3408 steps were used for 0.1, 0.32, 0.8, 1.5, 2.2, 2.9, 3.6, 4.3, 5 pN, respectively.

Figure 5A orange: 4564, 4425, 4469, 4515, 4593, 4396, 4327, 4295 steps were used for 0.1, 0.32, 0.8, 1.5, 2.2, 2.9, 3.6, 4.3, 5 pN, respectively.

Figure 5B red: 1022, 1064, 888, 763, 720, 839, 784, 881 steps were used for 0.1, 0.2, 0.3, 0.4, 0.7, 1, 1.3, 1.6, 1.9, 2.2 pN, respectively.

Figure 5B blue: 140, 128, 136, 144, 151, 144, 133, 136 steps were used for 0.1, 0.2, 0.3, 0.4, 0.7, 1, 1.3, 1.6, 1.9, 2.2 pN, respectively.

Figure 5B green: 74, 74, 72, 82, 83, 78, 74, 71 steps were used for 0.1, 0.2, 0.3, 0.4, 0.7, 1, 1.3, 1.6, 1.9, 2.2 pN, respectively.

Figure 5B orange: 545, 534, 544, 583, 574, 601, 567, 565 steps were used for 0.1, 0.2, 0.3, 0.4, 0.7, 1, 1.3, 1.6, 1.9, 2.2 pN, respectively.

Figure 5C red: A total of 6813 steps were used. These were binned as: 1464, 1220, 1005, 1076, 1205, 737, 106 steps were used for relative extension 0.31, 0.4, 0.5, 0.6, 0.7, 0.8, 0.9, respectively.

Figure 5C blue: A total of 12951 steps were used. These were binned as: 4400, 3011, 2053, 1647, 1221, 545, 74 steps were used for relative extension 0.31, 0.41, 0.5, 0.6, 0.7, 0.79, 0.89, respectively.

Figure 5C green: A total of 11849 steps were used. These were binned as: 2804, 2449, 1820, 1804, 1820, 1035, 117 steps were used for relative extension 0.3, 0.4, 0.5, 0.61, 0.71, 0.81, 0.91, respectively.

Figure 5C orange: A total of 12014 steps were used. These were binned as: 3221, 2139, 1761, 1733, 1801, 1156, 203 steps were used for relative extension 0.32, 0.41, 0.51, 0.6, 0.69, 0.78, 0.88, respectively.

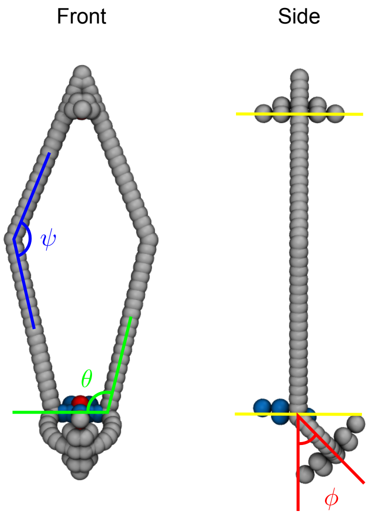

Fig. S1. Interaction angles controlling the geometry of the SMCC (see Table S2). Additional dihedral interactions were used to fix the relative orientation of the top binding site and the ATP bridge (yellow lines). These have no physical meaning and are hence not shown.

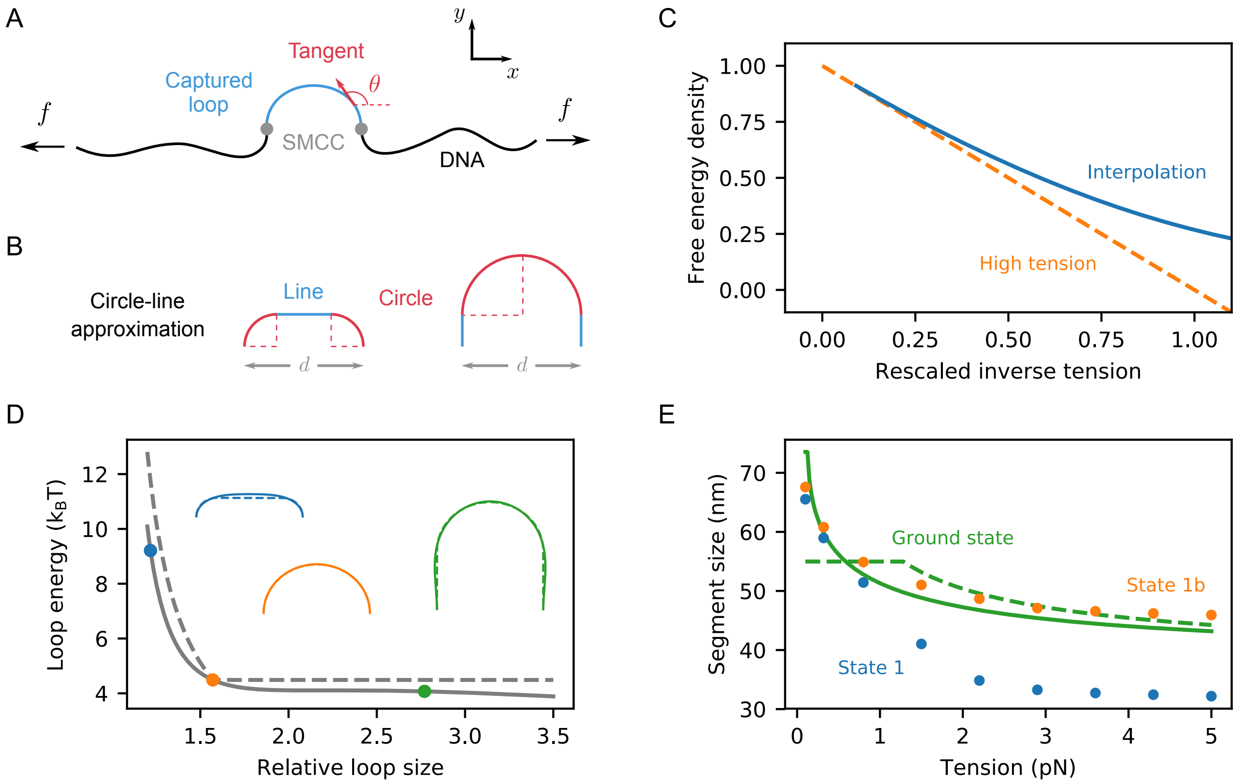

Fig. S2. Ground-state calculation of captured segment size by SMCC. (A) During the ATP-bound state of the cycle, the SMCC model (shown with gray) captures a loop (blue line, see also state 1b in Fig. 1C of main text), the size of which depends on the applied DNA tension, f. Mathematically, the loop can be parametrized with a single angle, θ, which denotes the orientation of the tangent to the loop curve (indicated with red). (B) The captured loop can be described by a circle-line approximation, which consists of either two quadrants connected with a line (left) or a semicircle extended with two lines (right), depending on the loop length relative to its end-point distance, d. (C) Normalized free energy per unit length, $-g(f)/f$, of the stretched (*i.e.,* nonlooped) DNA, as a function of the dimensionless quantity $(A\beta f)^{-1/2}$. The solid, blue line is a numerical calculation, based on the interpolation formula of Ref. 11, while the dashed, orange line is high-force approximation, given by $\mathrm{Eq.}\ (2)$. (D) Ground-state energy, $\varepsilon_{\text{loop}}$, *vs.* the relative loop size, L/d, together with the corresponding loop shapes for some selected values. The exact ground-state calculation [$\mathrm{Eq.}\ (7)$] is shown with solid green line, while the circle-line approximation [$\mathrm{Eq.}\ (4)$] with dashed green line. (E) Captured loop size *vs.* the applied DNA tension. Both the exact ground-state calculation (solid, green line) and the circle-line approximation (dashed, green line) seem to overestimate the simulation SMCC data (blue points) at high DNA tension. This deviation originates from partial-loop-capture events by the SMCC (state 1a in Fig. 1C of main text), which become increasingly probable at high tension. Excluding those from the calculation (orange points), reveals a good agreement with the ground-state theory.

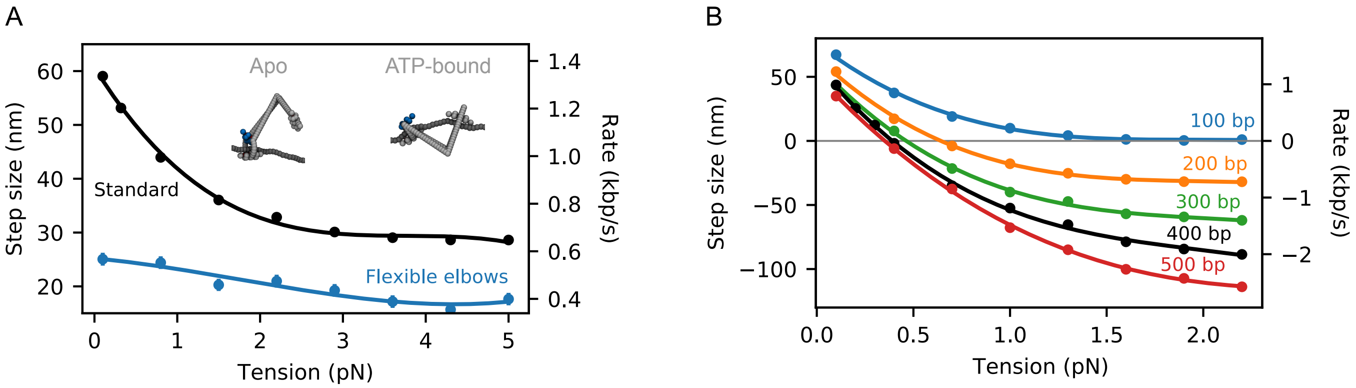

Fig. S3. (A) Translocation step size *vs.* applied DNA tension, for completely flexible SMC arms. When the elbow stiffness is suppressed, translocation slows down (blue points), and exhibits a weak dependence on the DNA tension. Inspection of the simulated configurations (attached snapshots) reveals that, this is due to the SMCC folding in the ATP-bound state, rather than DNA looping. For comparison, the data of Fig. 2 of main text are also shown with black points, corresponding to semiflexible SMC elbows. (B) Dependence of the loop extrusion step size *vs.* applied DNA tension on the initial loop size. For comparison, the data of Fig. 3A of main text are shown with black points.

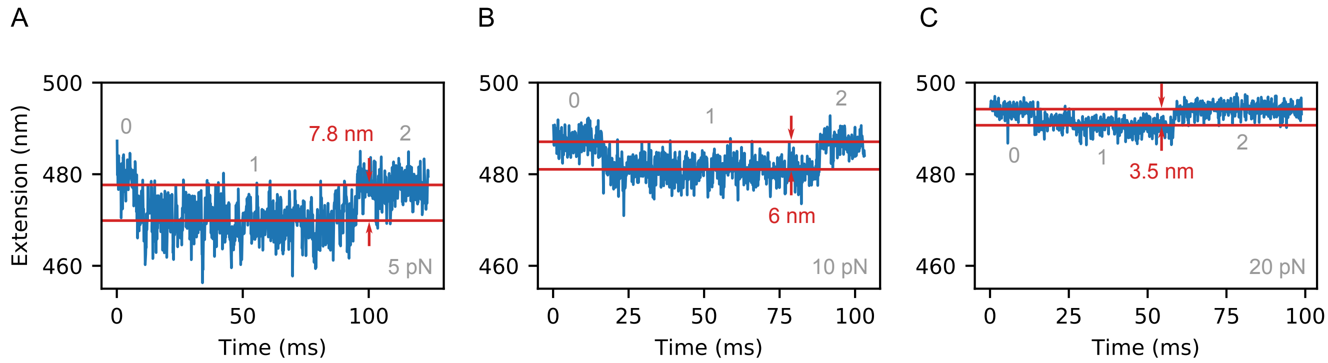

Fig. S4. Time traces of DNA extension for a translocation cycle by SMCC, at DNA tension of (A) 5 pN, (B) 10 pN and (C) 20 pN. In all cases, the ATP-bound state (1) of the SMCC brings about a noticeable DNA contraction (indicated with red), which is a signature of the segment-capture mechanism (state 1 in Fig. 1C,D of main text). As expected, this is a decreasing function of DNA tension, as segment capture becomes increasingly unfavorable. In order to convert from simulation to real time units, we used a mean cycle duration of 0.13 s, as estimated in Fig. 3 of main text.

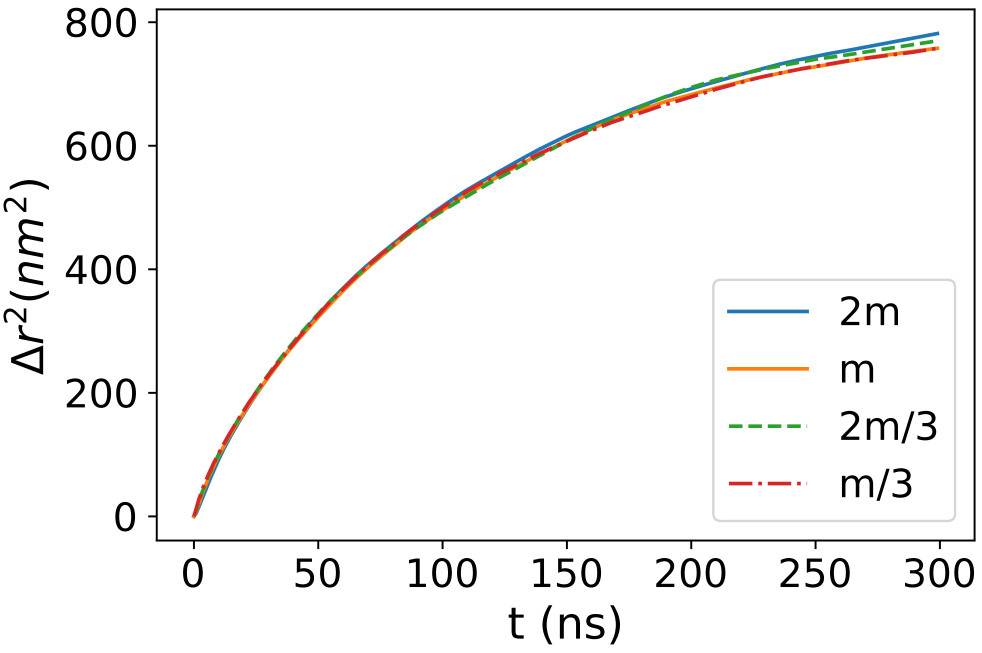

Fig. S5. Mean squared displacement of the central monomer of a 400 bp ($L = 136$ nm) coarse-grained DNA loop vs time. The end-points of the loop are kept fixed at a distance of 23 nm. Simulations were repeated for different values of the mass of the coarse-grained beads m and damping coefficient d_m, keeping the ratio m/d_m constant. The data reported as m represent simulations with the parameters as used in the main text. The overlap of the data show that intertial effects are negligible beyond the time scale of 1 ns.

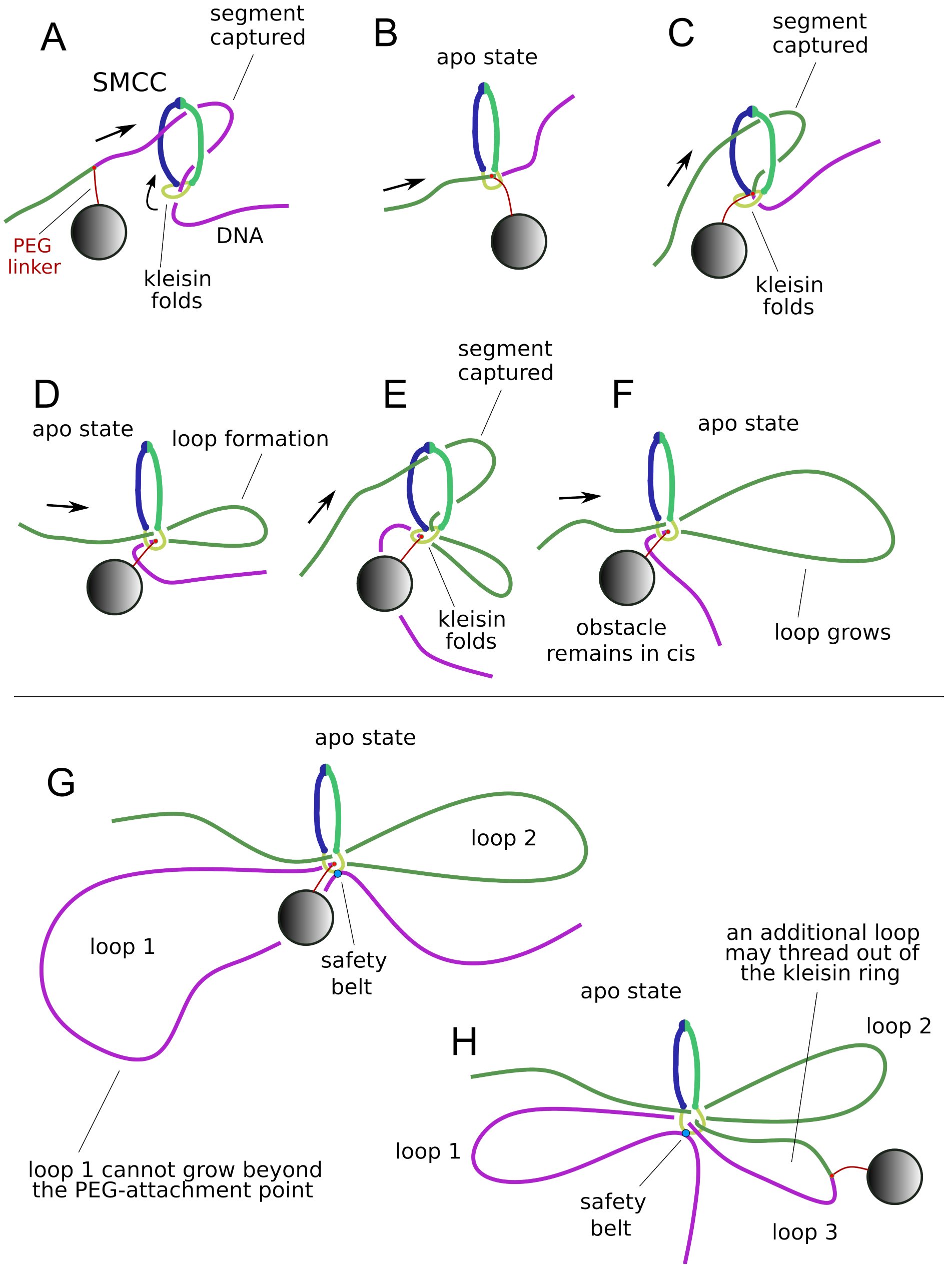

Fig. S6. Schematic illustration of the interaction of a SMCC with a DNA to which a large nanoparticle is attached via a PEG-linker as in recent experiments (16). The figure illustrates how the SMCC handles the obstacle in the segment-capture model. For clarity we used different colors to identify different parts of the DNA. (A-F): The translocation process proceeds normally via segment-capture (A) until the obstacle reaches the basis of the SMCC (B). The obstacle remains then topologically trapped in the vicinity of the SMCC while the segment-capture mechanism can continue to operate (C) inducing the formation of a loop (D). As the process continues, captured segments (E) contribute to the growth of the loop (F). (G-H): Loop extrusion with external safety-belt mechanism (only the apo state is shown here). The extrusion proceeds regularly until the obstacle reaches the base of the SMCC. From that point on a secondary loop is extruded (G), as in translocation (D). The loop 1 cannot grow beyond the PEG-attachment point, but loop 2 keeps growing following the cycles of segment-capture as illustrated in (E) and (F). Note that the nanoparticle/obstacle need not remain in the vicinity of the SMCC (H), but another loop can thread out of the kleisin ring (loop 3). Also the same scheme applies to the cases where the anchoring point is outside the tripartite SMCC ring (topological loading) or where it is inside the ring (pseudotopological loading).

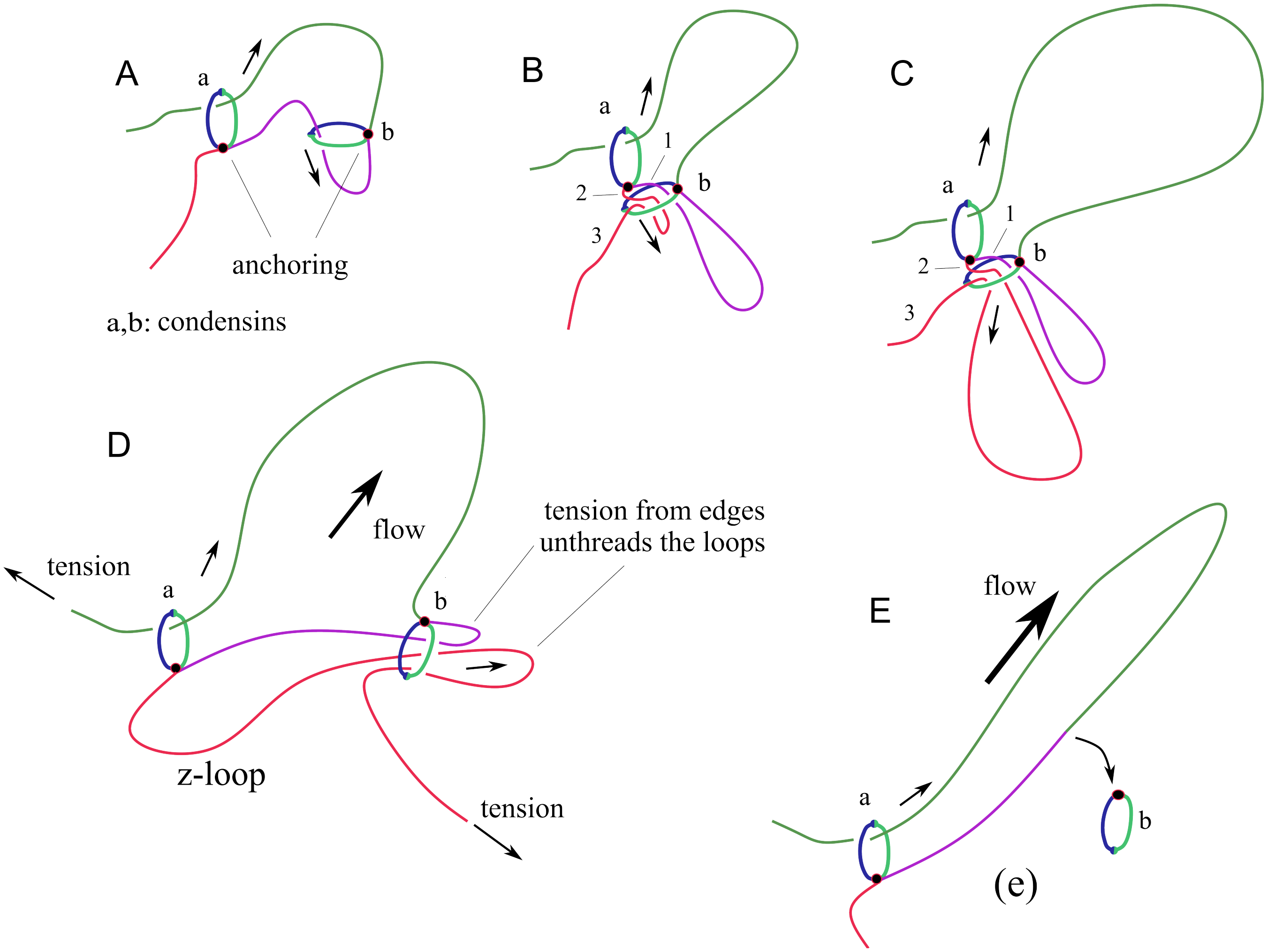

Fig. S7. Condensins extruding loops on DNA were shown to be able to traverse one other (17). The figure illustrates a possible explanation of the apparent "crossing" based on the segment-capture model. (A) Two nested condensins (a and b) extruding loops asymmetrically, where the black dots denotes the anchoring point. The arrows show the direction of motion of the translocating strands. (B) While extruding the purple loop the condensin b approaches condensin a. This approach can lead to partial threading of a through b so that the red strand can be captured by condensin b. Three strands (shown as 1, 2 and 3) are in the ring. (C) The segment capture mechanism cannot work on strand 1 and 2 if these are too short, therefore condensin b pulls primarily strand 3, enlarging the red loop. (D) As the two endpoints of the DNA are attached to a solid surface in the experiments of Ref. (17), some tension builds up at the edges. This induces a separation of the two condensins from each other, generating the characteristic "Z-loop" shape observed under flow condition in the experiments (17). (E) The increasing tension and flow may lead to the unthreading of the loops from condensin b, leaving a single loop through a, as observed in experiments (17). This process can occur in either the case where the anchoring sites indicated are outside the tripartite SMCC ring ("safety belt" mechanism with topological loading, thought to be the case for yeast condensin) or inside the SMCC ring ("pseudo-topological loading).

Movie S1. A typical translocation cycle for a DNA tension of 0.1 pN, showing the segment capture by the top binding site (state 1b).

Movie S2. A typical translocation cycle for a DNA tension of 10 pN, showing the "inchworm"-like motion of the SMCC (state 1a).

Movie S3. Gradual loop extrusion of DNA with fixed end points by SMCC (10 cycles).

References

1. M Hirano, T Hirano, Opening closed arms: long-distance activation of SMC ATPase by hinge-DNA interactions. *Mol Cell* **21**, 175–186 (2006).
2. RV Nunez, LBR Avila, S Gruber, Transient DNA occupancy of the SMC interarm space in prokaryotic condensin. *Mol. Cell* **75**, 209–223 (2019).
3. JJ Griese, G Witte, KP Hopfner, Structure and DNA binding activity of the mouse condensin hinge domain highlight common and diverse features of SMC proteins. *Nucleic Acids Res.* **38**, 3454–3465 (2010).
4. S Uchiyama, et al., Structural Basis for Dimer Formation of Human Condensin Structural Maintenance of Chromosome Proteins and Its Implications for Single-stranded DNA Recognition. *J. Biol. Chem.* **290**, 29461–29477 (2015).
5. S Datta, L Lecomte, CH Haering, Structural insights into DNA loop extrusion by SMC protein complexes. *Curr. Opin. Struct. Biol.* **65**, 102–109 (2020).
6. H Koide, N Kodera, S Bisht, S Takada, T Terakawa, Modeling of DNA binding to the condensin hinge domain using molecular dynamics simulations guided by atomic force microscopy. *bioRxiv* (2021).
7. Y Liu, et al., ATP-dependent DNA binding, unwinding, and resection by the Mre11/Rad50 complex. *EMBO J.* **35**, 743–758 (2016).
8. Y Li, et al., Structural basis for Scc3-dependent cohesin recruitment to chromatin. *eLife* **7**, e38356 (2018).
9. M Kschonsak, et al., Structural basis for a safety-belt mechanism that anchors condensin to chromosomes. *Cell* **171**, 588–600 (2017).
10. JF Marko, Biophysics of protein–DNA interactions and chromosome organization. *Phys. A* **418**, 126–153 (2015).
11. C Bouchiat, et al., Estimating the persistence length of a worm-like chain molecule from force-extension measurements. *Biophys. J.* **76**, 409–413 (1999).
12. I Kulić, H Schiessel, Nucleosome repositioning via loop formation. *Biophys. J.* **84**, 3197–3211 (2003).
13. S Sankararaman, JF Marko, Formation of loops in DNA under tension. *Phys. Rev. E* **71**, 021911 (2005).
14. SK Nomidis, et al., Twist-bend coupling, twist waves, and the shape of DNA loops. *Phys. Rev. E* **100**, 022402 (2019).
15. S Plimpton, Fast parallel algorithms for short-range molecular dynamics. *J. Comput. Phys.* **117**, 1–19 (1995).
16. B Pradhan, et al., Smc complexes can traverse physical roadblocks bigger than their ring size. *bioRxiv* (2021).
17. E Kim, J Kerssemakers, IA Shaltiel, CH Haering, C Dekker, DNA-loop extruding condensin complexes can traverse one another. *Nature* **579**, 438–442 (2020).
